# An Integrated Assessment of Different Depositional Paleoenvironment Using Nitrogen Markers and Biomarkers after Chromatographic Methods Optimization

**DOI:** 10.1021/acsomega.4c04189

**Published:** 2024-09-05

**Authors:** Flávia Lima e Cima Miranda, Diego Nery do Amaral, José Roberto Cerqueira, Karina Santos Garcia, Antônio Fernando de Souza Queiroz, Maria Elisabete Machado

**Affiliations:** † Programa de Pós graduação Geoquímica: Petróleo e Meio Ambiente, Instituto de Geociências, Universidade Federal da Bahia, Salvador 40170-290, BA, Brazil; ‡ Departamento de Química Analítica, Instituto de Química, Universidade Federal da Bahia, Salvador 40170-290, BA, Brazil; § Centro Interdisciplinar de Energia e Ambiente, Universidade Federal da Bahia, Salvador 40170-290, BA, Brazil; ∥ Departamento de Ciências Exatas, Universidade Estadual de Feira de Santana, Feira de Santana 44036-900, BA, Brazil

## Abstract

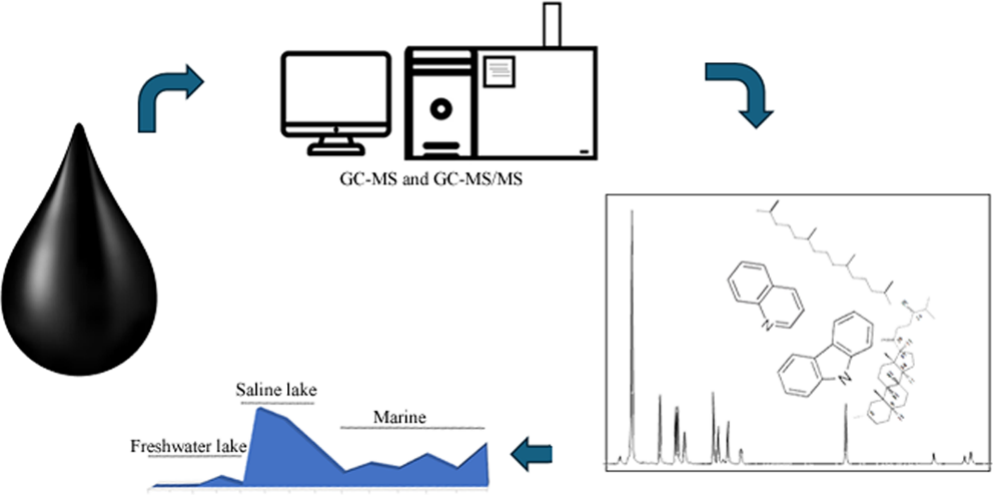

In this study, chromatographic methods were first optimized to ensure the robustness of the identification and quantification of nitrogen markers and biomarkers. Then, the optimal conditions were applied to 14 crude oil samples deposited in distinct paleoenvironments from Brazil, Venezuela, and Colombia to perform an integrated geochemical assessment. Analytical standards, certified reference material, and retention indices were used to confirm the identification of biomarkers and N-markers. The results of geochemical interpretations based on unresolved complex mixture (UCM), pristane/*n*-heptadecane (Pr/*n*-C_17_), and phytane/*n*-octadecane (Ph/*n*-C_18_) ratios and concentrations of carbazole and benzo[*b*]carbazole indicated that all oils are not biodegraded. The Pr/*n*-C_17_, Ph/*n*-C_18_, and Pr/Ph ratios showed that the organic matter that generated the oils from Brazil and Venezuela was deposited under anoxic conditions and Colombia oil reached dysoxic conditions. Some samples present a greater abundance of low- to high-molecular-mass *n*-alkanes, indicating freshwater lakes’ organic matter (Brazil oils). In contrast, other samples showed a lower abundance of high-mass *n*-alkanes, suggesting marine and saline lake origins (Colombia and Venezuela oils). The tricyclic/hopane ratio, the ternary diagrams using 1-methylcarbazole, 2-methylcarbazole, and 4-methylcarbazole, and regular steranes C_27_, C_28_, and C_29_ suggested a contribution of algae to the formation of kerogen present in the source rocks of all petroleum samples. The high concentrations of carbazole in oils generated by marine organic matter confirm the more positive δ^13^C values compared with those generated by lake organic matter (Brazil samples). The use of chemometric tools as principal component analysis exhibited a grouping of samples according to the depositional environment using carbazole and tricyclic/hopane ratio. The integration of all parameters analyzed provides a guide for refined interpretations and differentiation of oils according to their depositional environments.

## 1 Introduction

Crude oil is a complex mixture of organic compounds, including *n*-alkanes, steranes, terpanes, and isoprenoids. Target compounds of these classes are used as biomarkers in petroleum exploration to provide information on source material, nature of the depositional environment, thermal maturity, extent of biodegradation, and undertaking oil–oil and oil–source rock correlations.^1^ Low concentrations of nitrogen, sulfur, and oxygen are also present in crude oils, and specific organic compounds of these heteroatom classes, called markers, can provide information about petroleum similar to biomarkers.^2^

The formation of crude oil results from several chemical, biological, and physical transformations of organic matter preserved in sediments deposited in oxygen-deficient environments.^3^ The accumulation of organic matter occurs in different types of depositional environments such as fresh lakes, saline lakes, hypersaline, or marine. The organic matter undergoes alteration under varying physical-chemical conditions, mainly due to increased temperature, resulting in oil formation with different chemical characteristics.^3,4^

Biomarkers play a crucial role in organic geochemistry, offering insights into the origin, migration, source, accumulation, biodegradation, environmental conditions during deposition of their source rocks (diagenesis), thermal maturity (catagenesis), and lithology.^2,5^*n*-Alkanes are widely used biomarkers due to their little structural change in the oil formation process.^2,6^ The number of carbons in the *n*-alkane structure allows the evaluation of the type of organic matter present in the depositional paleoenvironment of the hydrocarbon source rocks.^2,4^ Hopanes are another class of biomarkers that, due to stereochemistry, form several other compounds in relative abundance. This allows the degree of thermal evolution or level of biodegradation of oils to be estimated.^7^ Steranes are used to provide information about the depositional paleoenvironment of source rocks, to characterize and evaluate the sources of organic matter between marine and terrigenous and thermal evolution.^2^

Geochemical markers such as nitrogen compounds (N-markers) are found at low concentrations in crude oils. N-markers are used to evaluate oil origin, organic facies, maturation, and depositional paleoenvironment of hydrocarbon source rocks.^8^ For example, carbazoles (CA) and benzocabazoles (BCA) are in greater abundance in oils generated by marine organic matter when compared to those originated by lacustrine organic matter.^9−12^ Ternary diagrams using 1-methylcarbazole (1MCA), 2-methylcarbazole (2MCA), and 4-methylcarbazole (4MCA) or C_27_, C_28_, C_29_ steranes and N-markers provide information about organic matter from marine or terrestrial origin.^13^

Contents of sulfur (S) and the isotopic ratio δ^13^C of organic carbon have been employed as tools in evaluating the depositional paleoenvironment of hydrocarbon source rock.^6,14^ The S % delineates the depositional environment and its oxygenation conditions because it results from the reduction of the sulfate ion present in greater quantities in marine waters compared to continental waters.^15^ The δ^13^C isotopic ratio of organic carbon in petroleum provides a historical record of the different sources of OM and the physicochemical conditions of the depositional paleoenvironment.^16^ Isotopes of carbon sources are produced to distinguish between oils from marine and nonmarine sources.^6^ Isotopes of organic carbon from shales can indicate contributions of organic matter of marine or continental origin. OM present in freshwater lake environments has δ^13^C values lower than −28‰, enriched in ^12^C, while in saline environments, due to the relative enrichment in ^13^C, δ^13^C values are higher than −28‰.^17−19^

Gas chromatography (GC) coupled to mass spectrometry (MS) and tandem MS (MS-MS) are the most common techniques used to evaluate biomarkers and markers for geochemical characterization. Despite the well-known capabilities of these techniques, there are certain limitations, such as method optimization, the use of authentic standards, and quantification, that are not performed. The description of this step, in general, is limited to identification based on the retention time of previous studies and compound area. In some cases, not even the retention times are indicated in chromatograms, which makes accurate and reliable identification difficult. Thus, there is a lack of studies in organic geochemistry with criteria and detailed information about the correct determination and quantification of organic biomarkers and markers.

In this study, chromatographic methods were optimized to ensure the robustness of the identification and quantification of biomarkers and N-markers. The optimal method conditions were applied to 14 crude oil samples deposited in distinct paleoenvironments (freshwater lake, saline lake, and marine) to perform an integrated geochemical assessment. Principal component analysis (PCA) and hierarchical cluster analysis (HCA) tools were employed to explore similarities and hidden patterns among the samples. Stable isotopes of organic carbon and total sulfur were also used to evaluate the conditions of the sedimentary paleoenvironment of the source rocks of the oil samples.

## 2 Materials and Methods

### 2.1 Samples

**Figure 1:**
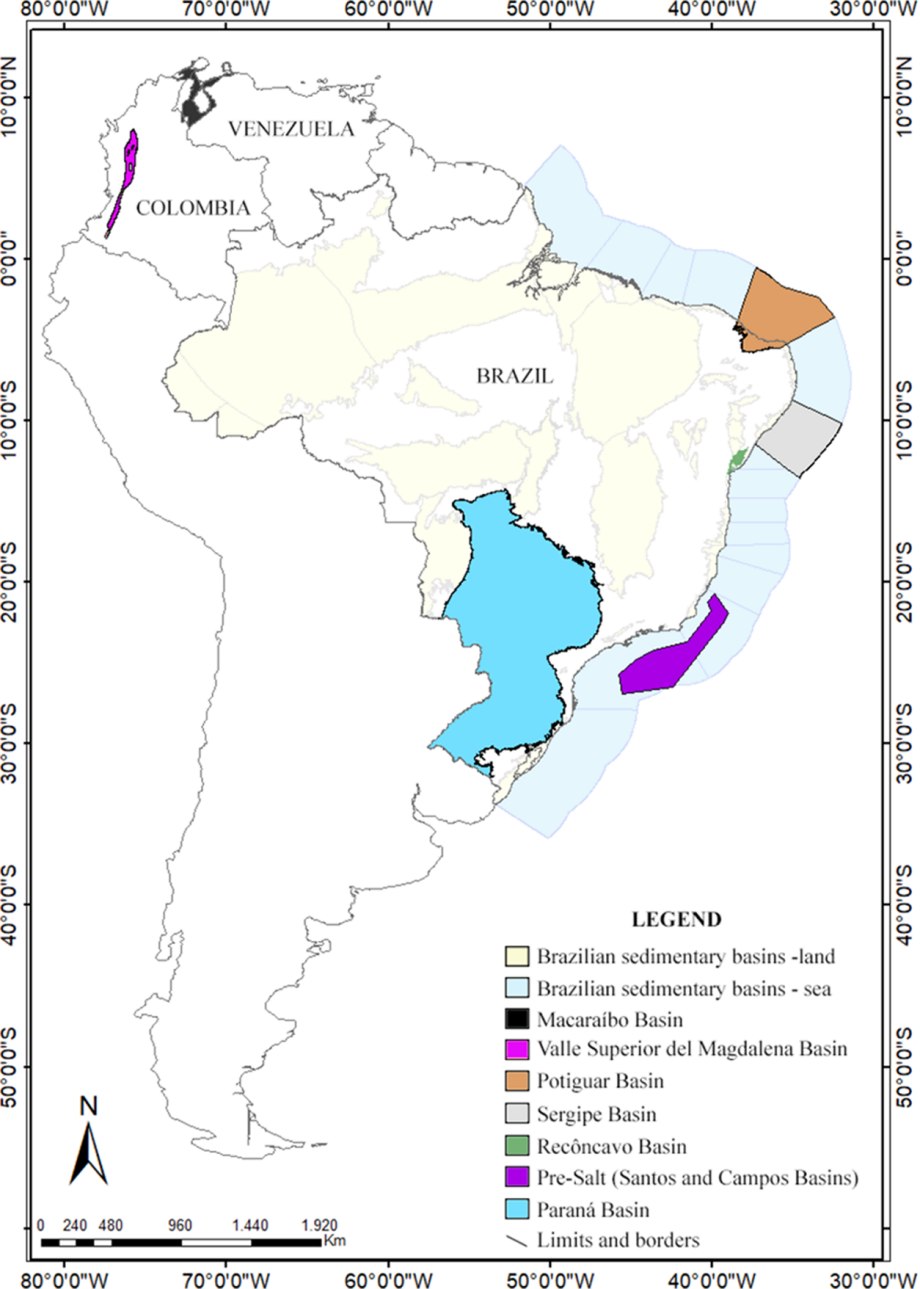
Map showing the localization of the basins of the oil samples evaluated.

Fourteen crude oil samples from different sedimentary basins of Brazil, Venezuela, and Colombia were obtained from the inventories of the Lepetro Laboratory (Institute of Geoscience, Federal University of Bahia, Brazil). The samples were selected based on depositional paleoenvironments of their source rocks (freshwater and saline lakes, hypersaline, and marines), as demonstrated in Table 1. The map with the region location of samples is shown in Figure 1. The petroleum fields where the samples were collected were omitted due to the confidential nature of this information.

**Table 1:** Crude Oil Samples According to Environment Depositional Type and Respective Basins

basin	environment type	identification	refs
Recôncavo, Brazil	freshwater lake	REC 1	(20)
		REC 2	
Potiguar, Brazil	freshwater lake/marine	POT 1	(20)
		POT 2	
Campos, Brazil	saline lake	3MLL	(21)
		6CHT	
Paraná, Brazil	hypersaline/marine	PAR 1	(22)
		PAR 2	
Santos, Brazil	saline lake	9LL	(21)
Sergipe, Brazil	freshwater lake/saline lake/marine	SERG	(23)
Maracaibo, Venezuela	marine	VEN 1	(39)
		VEN 2	
Valle superior del Magdalena, Colombia	marine	COL 1	(24)
		COL 2	

#### 2.1.1 Stratigraphic Information

In the Recôncavo Basin, the Candeias Formation (Berriasian) represents the initial phase of deposition in an aborted intracontinental rift (aulacogenic), deposited in a freshwater lacustrine context in the Cretaceous period.^20^

In the Potiguar Basin, there is evidence of evolution with different phases (Rift I, Rift II, Postrift, and Drift) associated with the South Atlantic Rift. Due to these characteristics, the Potiguar Basin can present different types of source rocks (deposited in the Cretaceous) and, consequently, oils with different characteristics: lacustrine (Pendência Formation; Rift I phase, Berriasian to Berremian) and marine (Alagamar Formation; Post Rift phase, Aptian).^20^

In the Sergipe-Alagoas Basin, there are different phases of evolution associated with the South Atlantic Rift: Pré-Rifte, Rifte, Pós-Rifte e Drifte. The Barra de Itiba and Coqueiro Seco Formations (Hauterivian to Aptian) represent the lacustrine rift interval. The Maceió Formation (Aptian) represents the saline lake rift phase in the Basin. The Muribeca formation was deposited in a marine context developed at the end of the Aptian, marking the postrift phase of the Basin.^23^

The main source rock of the Campos Basin is shales deposited in a lacustrine context from the Lagoa Feia Group (Barremian/Aptian), but there are still intervals with possible marine influences, generating a lake with salty waters. In Santos Basin are two intervals that generate hydrocarbons, namely, the Piçarras Formation, deposited in a saline lacustrine context in the final stage of the Rift phase, in the Aptian, and the Itajaí-Açu Formation, represented by shales and dark gray mudstones deposited in the environment platform.^21^

The Paraná Basin’s source rocks are represented by Irati and Ponta Grossa Formations; in both, the formation of hydrocarbons occurs from the thermal effect of intrusive rocks in contact with shales rich in organic matter. The Irati Formation is predominantly characterized by carbonates and evaporites deposited in the Permian, in a lacustrine context, and sometimes under hypersaline environmental conditions. The Ponta Grossa Formation of the Devonian age is characterized by black shales deposited in a marine context.^22^

La Luna Formation is the source rock of the Maracaibo Basin, Venezuela, and Valle Superior del Magdalena Basin, Colombia. The accumulation of organic matter on this basis occurred in an anoxic environment in western Venezuela and a small part of eastern Colombia during the Cretaceous period (Cenomanian – Campanian).^24,39^

### 2.2 Reagents and Standards

Silica gel (pore size 60 Å, particle diameter 63–200 μm) was purchased from Sigma-Aldrich (St. Louis). The solvents dichloromethane (DCM), isopropanol, methanol (MeOH), and n-hexane were spectroscopic/HPLC grade and purchased from Merck (Darmstadt, Germany).

Thirty-one individual standards of biomarkers and markers and five internal standards (IS) were employed for the chromatographic method optimization and quantification. *n*-Alkanes (C_7_-C_40_), pristane, and phytane were sourced from Sigma-Aldrich (St. Louis, MO). The IS eicosane-d_42_ (*n*-C_42_d), *n*-octacosane-d_58_ (*n*-C_58_d), *n*-hexadecane-d_34_ (*n*-C_34_d), 5β-cholane and the standards C_27_17α-hopane (Tm), 21β-22,29,30-Trisnorhopane, C_30_17β,21α-hopane, 18β(H)-oleanane, and Gammacerane were purchased from Chiron AS (Trondheim, Norway). The N-markers: indole (IND), 3-methylindole (3-MIND), carbazole (CA), quinoline (QUIN), 4-methylquinoline (4-MQUIN), 2,4-dimethylquinoline (2,4-DMQUIN), benzo[*c*]quinoline (B[*c*]QUIN), acridine (ACR) 1-methylcarbazole (1-MCA), 2-methylcarbazole (2-MCA), 9-methylcarbazole (9-MCA), 1,8-dimethylcarbazole (1,8-DMCA), 2,7-dimethylcarbazole (2,7-DMCA), 3,6-dimethylcarbazole (3,6-DMCA), 3-ethylcarbazole (3-EtCA), 1,4,8-trimethylcarbazole (1,4,8-TMCA), benzo[*a*]carbazole (B[*a*]CA), benzo[*b*]carbazole(B[*b*]CA), benzo[*c*]carbazole (B[*c*]CA), dibenzocarbazole (DBCA), and also the IS carbazole-d8 (CA-d_8_) and 9-phenylcarbazole (9-PCA) were acquired from Chiron AS (Trondheim, Norway).

Standard Reference Material (SRM) of the oil from the Gulf of Mexico SRM 2779 (NIST, Gaithersburg, Maryland) was employed to identify and confirm the biomarkers.

### 2.3 Total Petroleum Hydrocarbon Analysis

Gas chromatographic analyses of the TPH in oil were performed on an Agilent 7890B (Wilmington) instrument equipped with a split/splitless injector. The samples, dissolved in DCM at 0.05 mg μL^–1^, were injected (1 μL) into the autosampler at 300 °C in splitless mode. The separation was performed on a 100% dimethylpolysiloxane (DB-1) capillary column (15 m × 0.25 mm × 0.25 μm) using hydrogen (1 mL min^-1^) as carrier gas. The oven temperature was set to 40 °C (2 min) and heated at 10 °C min^–1^ to 300 °C where it was held for 12 min. The detector temperature was 300 °C.^25^

The aim of TPH analysis was to infer the quality of the organic matter that originated the oil and also to obtain information about thermal maturity and degree of biodegradation by evaluation of the chromatogram profile of the unresolved complex mixture (UCM).

### 2.4 Sara Fractionation

Oils and the SRM 2779 were fractionated using the SARA method^26^ and separated into saturated, aromatic, and NSO fractions. The saturate fraction was used to determine the biomarkers (hopanes and steranes), and the aromatic and NSO fractions were used to determine the N-markers.

Glass column columns (30 cm high × 0.2 cm diameter) were filled with silica gel (0.063–0.200 mm, Sigma-Aldrich, St. Louis) previously activated at 450 °C for 4 h. Silica premoistened with n-hexane was used to pack the column to a height of 12 cm. Approximately 20 mg of samples were added to the top of the column. The aliphatic fraction was eluted with 30 mL of *n*-hexane, the aromatic and NSO fraction with 40 mL of *n*-hexane/DCM (4:1, v:v) and 40 mL of DCM/MeOH (4:1, v:v), respectively. All extracts were concentrated in a rotary evaporator (Model R-210 Labortechnik, AG Switzerland) prior to chromatographic analysis.

### 2.5 Chromatographic Analysis

Chromatographic analyses for the determination of *n*-alkanes, hopanes, steranes, and N-markers were carried out on a GC-MS/MS system composed of an Agilent 7890B gas chromatograph equipped with a G4513A autoinjector, a split-splitless injector, and a mass triple quadrupole 7000C (Agilent Technologies, Palo Alto, CA). Data were acquired using MassHunter ver. B.07.00 (Agilent, CA). The MS library used was NIST version 2.7. Ultrapure helium gas was used as the carrier gas at a constant flow rate of 1.2 mL min^–1^, and 1 μL of the sample was injected in splitless mode with an injector temperature of 300 °C. A 5% phenyl and 95% dimethylpolysiloxane (DB-5MS) capillary column (30 m × 0.25 mm ID × 0.25 μm d_f_, Agilent, Santa Clara, CA) was used for separation.

The chromatographic conditions for individual biomarkers and markers were optimized using a set of analytical tools, such as analytical standards, SRM, retention index (RI), comparison of mass spectrum similarity, and retention time of similar works described in the literature. These method optimizations are described in the following items.

#### 2.5.1 Biomarkers Optimization

##### 2.5.1.1 *n*-Alkanes and Isoprenoids

To optimize the chromatographic method for *n*-alkanes, pristane, and phytane isoprenoids, a standard mix solution containing these compounds and the IS eicosane-d_42_, *n*-octacosane-d_58_, and *n*-hexadecane-d_34_ was prepared in a concentration of 100 μg L^–1^.

Quantification was carried out by the internal standards method (eqs 1 and 2) using the *n*-eicosane d_42_, *n*-octacosane d_58_, and *n*-hexadecane d_34_ standards at a concentration of 1000 μg L^–1^ in samples. The data processing was in extracted ion mode (EIM) using peak areas of *m*/*z* 57, *m*/*z* 66, *m*/*z* 71, *m*/*z* 85, and *m*/*z* 183 for quantification.
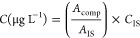
1where *C* = concentration, *A*_comp_ = area of the compound, *A*_IS_ = internal standard area, *C*_IS_ = internal standard concentration.

To convert the μg L^–1^ to μg g^–1^:
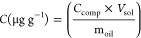
2where *C* = concentration, *C*_comp_ = compound concentration (μg L^–1^), *V*_sol_ = volume of solvent, *m*_oil_ = oil mass

##### 2.5.1.2 Hopanes and Steranes

To chromatographic method optimization to hopanes and steranes, a mixed standard solution containing C_27_ 17α, 21β-22, 29, 30-trisnorhopane, C_30_ 17β, 21α-hopane, 18β(H)-oleanane, 5β-colane and gammacerane were prepared at a concentration of 100 μg L^–1^. In addition, the SRM 2779 was employed to identify 17α(H),21β(H)-30-norhopane, 17α(H)-22,29,30-trisnorhopane, 18α(H)-22,29,30-trisnorhopane, 17α(H),21β(H)-30-hopane, 17α(H),21β(H)-22R-homohopane, 17α(H),21β(H)-22S-homohopane, 17α(H)-diahopane, 5α(H), 14β(H),17β(H)-cholestane 20S, 5α(H), 14β(H) and 17β(H)-cholestane 20R. RI also was used as confirmation criteria for the positive identification to increase the number of compounds identified (Item 3.1.2).

The quantification of hopanes and steranes was carried out by the IS method using 5β-cholane added at a concentration of 100 μg L^–1^ to the saturated fraction. The peak areas were used to determine the concentrations (eqs 1 and 2).

##### 2.5.1.3 Retention Index

Retention indices (RI) were calculated for each compound according to Kováts indices^27^ using the following equation:
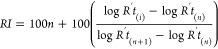
3where *n* = number of carbons in the least retained adjacent pattern, *R’t*_*(i)*_ = adjusted retention time of the analyte, *R’t*_*(n)*_ = retention time of the reference *n*-alkanes elute before analyte, *R’t*_*(n*+1*)*_ = retention times of the reference *n*-alkanes elute after the analyte.

RI was experimentally obtained using a standard mixture of *n*-alkanes (C_7_-C_40_) as external references and compared with those reported in the literature (NIST Mass Spectral Library) to DB-5 column (5% phenyl–95% methylpolysiloxane). To identify a compound, the difference between the experimental RI and the literature RI of 10 units was selected.

#### 2.5.2 Nitrogen Markers

The determination of N-markers in samples was carried out in MRM mode according to Dias et al., 2021. Briefly, the N-markers standards (described in Section 2.2) and samples were injected in splitless mode. The column oven temperature started at 80 °C (1 min), increased to 160 °C at a rate of 6 °C min, then to 200 °C at 20 °C, and increased at 4 °C/min to 280 at 310 °C (2 min). The MS operated in MRM mode at 70 eV.

Quantification of N-markers was performed by the IS method using 9-PhenCA and CA-d_8_ added in samples at 10 μg L^–1^ (eqs 1 and 2). N-marker concentrations were determined by using the most abundant MRM transition.

### 2.6 Organic Carbon Stable Isotopes Analysis

To provide robustness to the paleoenvironmental interpretation, the stable isotopes of organic carbon in the petroleum samples were determined using an elemental analyzer coupled to an isotope ratio mass spectrometer (EA-IRMS, Thermo Scientific EA IsoLink IRMS system, Bremen, Germany). A mass of crude oil sample between 0.4 and 0.6 mg was weighed into tin capsules (8 mm × 5 mm, Thermo Scientific) using an AUY220 precision analytical balance (Shimadzu, Kyoto, Japan) with minimum and maximum weighing limits 0.01 and 220 g, respectively. Stable carbon isotope ratios (*R* = ^13^C/^12^C) were expressed relative to the VPDB standard using “delta” notation, where: δ^13^C = (Sample/*R*_standard_ - 1) × 1000 (units are ‰ or per thousand or parts per thousand).

In the elemental analyzer, each sample was quantitatively burned with oxygen (99.9999% purity; Air Liquid, Brazil) added to the helium stream at a temperature of 1020 °C in a reactor composed of Cr_2_O_3_ (Thermo Scientific, Germany) and silver cobalt oxides (Thermo Scientific, Germany). The gases obtained were conducted through a water trap (Magnesium Perchlorate, Thermo Scientific, Germany) and separated on a Porapak Q 80/100 mesh (0.6 m × 1/8 in) packed column at 60 °C in an oven. Through a He stream (99.999% purity; Air Liquid, Brazil), CO_2_ was introduced into the isotope ratio mass spectrometer. The total race time was 300 s.

Carbon isotopic composition was calibrated against the VPDB scale using an NBS 22. The measurement uncertainty was monitored using 14 readings of the standard with well-characterized isotopic composition NBS 22 (bomb oil, δ^13^C – 30.03 ± 0.04‰). Accuracy was determined based on repeated measurements of the calibration standard and sample replicates. The analyses were divided into blocks of 12 readings and started and ended with the NBS 22 standard. The determined isotopic value was corrected by the average value of the standard. In the middle of the block, an NBS 22 standard capsule was inserted, which was used as a verification standard. Reproducibility was better than ± 0.1‰ for δ^13^Corg.^25^ The correction was calculated according to the following equations:
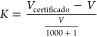
4
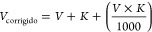
5where *K* = correction factor, *V* = raw isotopic value (determined in IRMS before correction), *V*_certificate_ = certified isotopic value of the NBS22 standard, *V*_corrected_ = the isotopic value of the sample corrected after correction.

### 2.7 Total Sulfur Analysis

Total sulfur analyses were performed in order to estimate the sulfur content for each sample. This parameter is important to assess the depositional environment and oxygenation conditions.^15^

The total sulfur content in crude oils was determined using a LECO 628 S (LECO Corporation) elemental analyzer operated at a maximum temperature of 1350 °C using approximately 0.1 g of samples and oxygen 5.0 (purity of 99.999%). The calibration for sulfur was performed using the CHNS standard (LECO).

### 2.8 Statistical Analysis

Principal component analysis (PCA) was used to evaluate the grouping of the oil samples according to the respective depositional environments of the organic material of origin. Hierarchical cluster analysis (HCA) aimed to classify samples based on similarities.

Statistical analysis of geochemical data was performed using R software for data processing (R Core Team, 2013). A correlation matrix of 14 samples by 3 parameters (14 × 3) was constructed to generate linear combinations of all variables.

## 3 Results and Discussion

### 3.1 Chromatographic Methods for Biomarkers

#### 3.1.1 *n*-Alkanes and Isoprenoids

The conditions of the optimized GC-MS method for *n*-alkane and isoprenoids were injection in splitless mode with the injector at 300 °C, column oven temperature starting at 40 °C, increasing at a rate of 4 °C min^–1^ until 300 °C, and remaining at this temperature for 15 min. The MS was operated in EI mode at 70 eV in full scan mode (*m*/*z* 40–450). The transfer line and ionization source temperatures were at 315 °C, and a He flow rate of 1.5 mL min^–1^.

**Figure 2:**
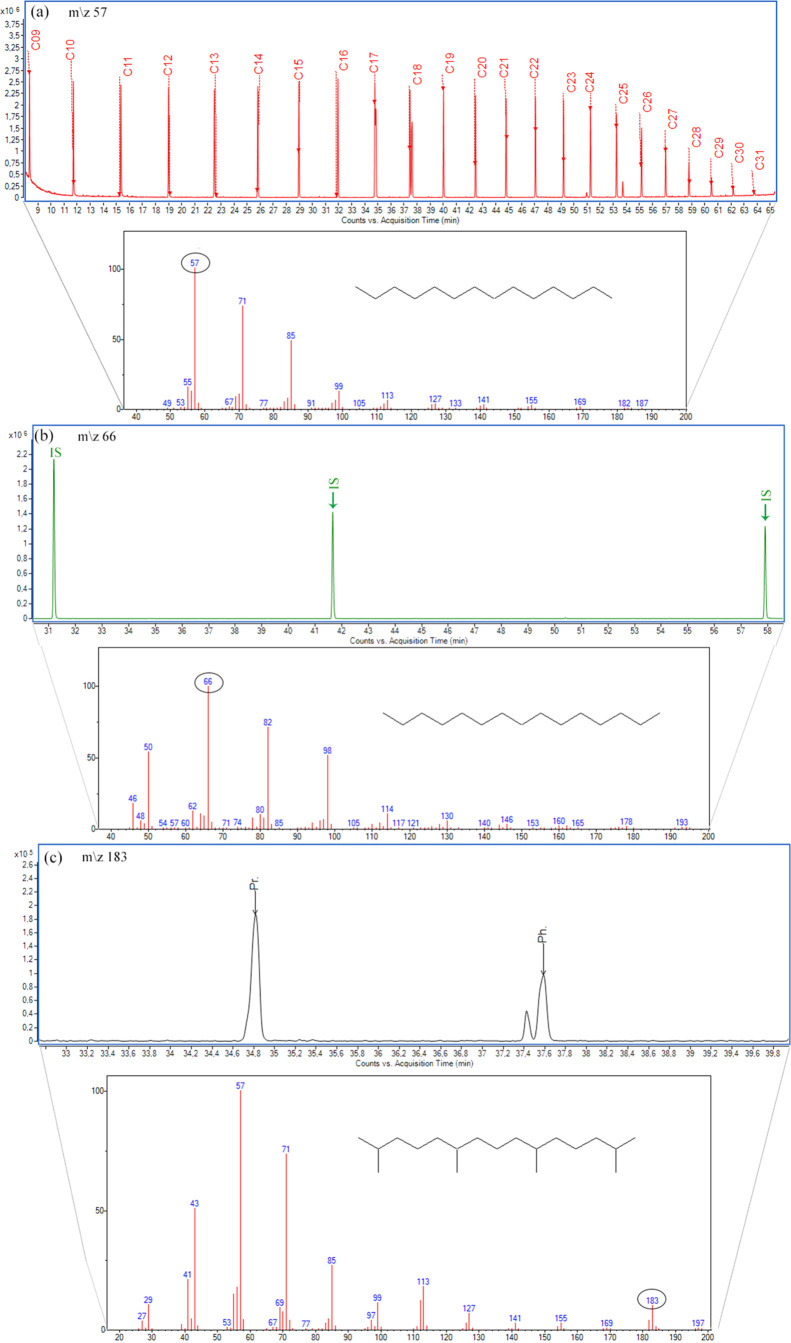
Chromatogram and mass spectrum for *n*-alkanes (a), internal standards (b), and isoprenoid (c)

All of the *n*-alkanes were identified by comparison with retention times and mass spectra of reference *n*-alkane standards (C_7_–C_40_) as shown in Figure 2a. For the quantification of compounds in samples, the chromatograms of the EIM signals by *m*/*z* values of 57 (*n*-alkanes), 66 for IS (Figure 2b), and 183 for identification of isoprenoids pristane and phytane (Figure 2c). The optimized method was applied in samples and allowed the identification (Table S1) and quantification (Table S2) of *n*-alkanes from C_9_ to C_31_. Several geochemistry studies employ ion *m*/*z* 57 for *n*-alkanes and isoprenoids. However, the use of this ion fragment can affect the selectivity, because the retention time for both compounds (*n*-alkanes and isoprenoids) is very similar in 5% phenyl columns (e.g., DB5MS, HP5). Thus, the ion more indicated for pristane and phytane is *m*/*z* 183.

#### 3.1.2 Hopanes and Steranes

The number of hopane and sterane standards commercially available is very limited. Hence, in geochemistry studies in general, the identification of these biomarkers is only tentatively done by mass spectra comparison and/or elution order in the chromatogram. To overcome these difficulties and increase reliability in compound identification, the use of SRM and Kováts IR were employed.

**Figure 3:**
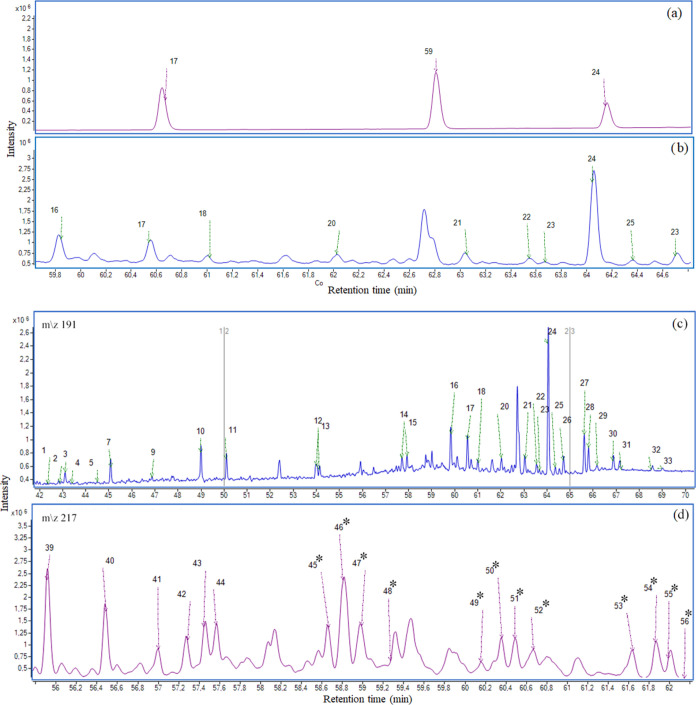
Chromatogram in SIM mode for standards of hopanes (a) and steranes (b). Chromatogram of hopanes (c) and steranes (d) identified in SRM 2779. *compounds identified by RI (for identification of the numbers, see Table S3).

The GC conditions were the same as those optimized to *n*-alkanes. For the MS, the SCAN mode was initially employed to obtain the retention time and mass spectrum of hopanes and steranes standard (described in 2.2 item). Afterward, the SIM mode monitoring the ions *m*/*z* 191 for hopanes and *m*/*z* 217 for steranes was used for quantification. The chromatograms of steranes and hopanes identified with standards and in SRM 2779 are shown in Figure 3a–d. To increase the number and reliability of compounds identified, Kóvatz RI was calculated, and this tool allowed the identification of 15 individual hopanes and steranes biomarkers (Table 2).

**Table 2:** Hopanes and Steranes Identified by Retention Index^a^

class	compound	*T*_R_ (min)	R.I_calculated_	R.I._literature_	refs
hopanes	T25	45.08	2108	2115	(28)
	T20	42.8	2019	2018	(28)
	H29	63.03	3058	3058	(28)
	M30	64.71	3167	3164	(29)
	H31(S)	65.61	3226	3229	(29)
steranes	C27 (R)	5866	2797	2797	(30)
	C27 (S)	59.32	2838	2836	(30)
	C27 αββ(R)	58.82	2806	2806	(30)
	C27 αββ(S)	58.97	2814	2815	(30)
	C28 (R)	61.10	2948	2941	(30)
	C28 (S)	60.35	2896	2896	(30)
	C28 αββ(R)	60.49	2909	2904	(30)
	C28 αββ(S)	60.63	2917	2912	(30)
	C29 (R)	62.69	3040	3037	(30)
	C29 (S)	61.63	2978	2972	(30)
	C29 αββ(R)	61.87	2986	2996	(30)
	C29 αββ(S)	62.01	2995	3001	(30)

a T_25_: C_25_ tricyclic terpane (a); T_20_: C_20_ tricyclic terpane; H_29_: 17α(H).21 β(H)-30-norhopane; M30: 17 β(H),21a(H)-hopane (moretane); H3(S): 22S-17a(H),21 β(H)-30-homohopane; C27(R): C27 20R-5a(H), 14a(H), 17a(H)-cholestane; C_27_(S): C_27_ 20S-5α(H), 14a(H), 17a(H)-cholestane; C_27_ αββ(R): C_27_ 20R −5α(H), 14β(H), 17∼(H)-cholestane; C_27_ αββ(S): CZ7 20S-5α(H), 14β(H), 17β(H)-cholestane; C_28_(R): C_28_ 20R-5α(H), 14a(H), 17a(H)-ergostane; C_28_(S): C_28_ 20S-5α(H), 14a(H), 17a(H)-ergostane; C_28_ αββ(R): C_28_ 20R-5α(H), 14β(H), 17β(H)-ergostane; C_28_ αββ(S): C_28_ 20S-5a(H), 14β(H),17β(H)-ergostane; C_29_(R): C_29_20R-5α(H), 14a(H), 17a(H)-stigrmastane; C_29_(S): C_29_ 20S-5α(H), 14u(H), 17a(H)-stigrmastane; C_29_ αββ(R): C_29_ 20R-5α(H), 14β(H), 17β(H)-stigmastane; C_29_ αββ(S): C_29_ 20S-5α(H), 14β(H), 17β(H)-stigrmastane

The quantification of individual hopanes and steranes in all samples was applied considering the identification tools described, and the results are shown in Table S4.

Authentic standards are necessary to determine compounds by GC-MS when quantification and reliable work are desired. Numerous compounds are employed for the class of hopane and sterane biomarkers in geochemistry studies. However, commercial standards are insufficient for the complete identification of all of the biomarkers employed in geochemistry studies. For this reason, the identification of compounds is typically based on mass spectral data comparison of GC retention data with the literature. The use of Kóvats RI and standards is very scarce.^31,32^

The use of SRM 2779 contributed to confirming the order of elution and subsequent identification and quantification of biomarkers, allowing for the paleoenvironmental reconstruction of all samples under study. The SRM is a crude oil from the Gulf of Mexico related to source rocks deposited in a mainly marine environment (in the Jurassic to Cretaceous period), followed by deposition with a more terrigenous influence throughout the Paleogene, but still a strong marine influence.^33^

### 3.2 Geochemical Interpretations

#### 3.2.1 Biodegradation and Thermal Maturation

The presence of UCM in the chromatograms of crude oil samples may indicate biodegradation. Oil biodegraded generally presents UCM with removal of *n*-alkanes in the high molecular weight range (C_20_-C_40_) or in the low molecular weight range (C_6_-C_12_).^2,34^

**Figure 4:**
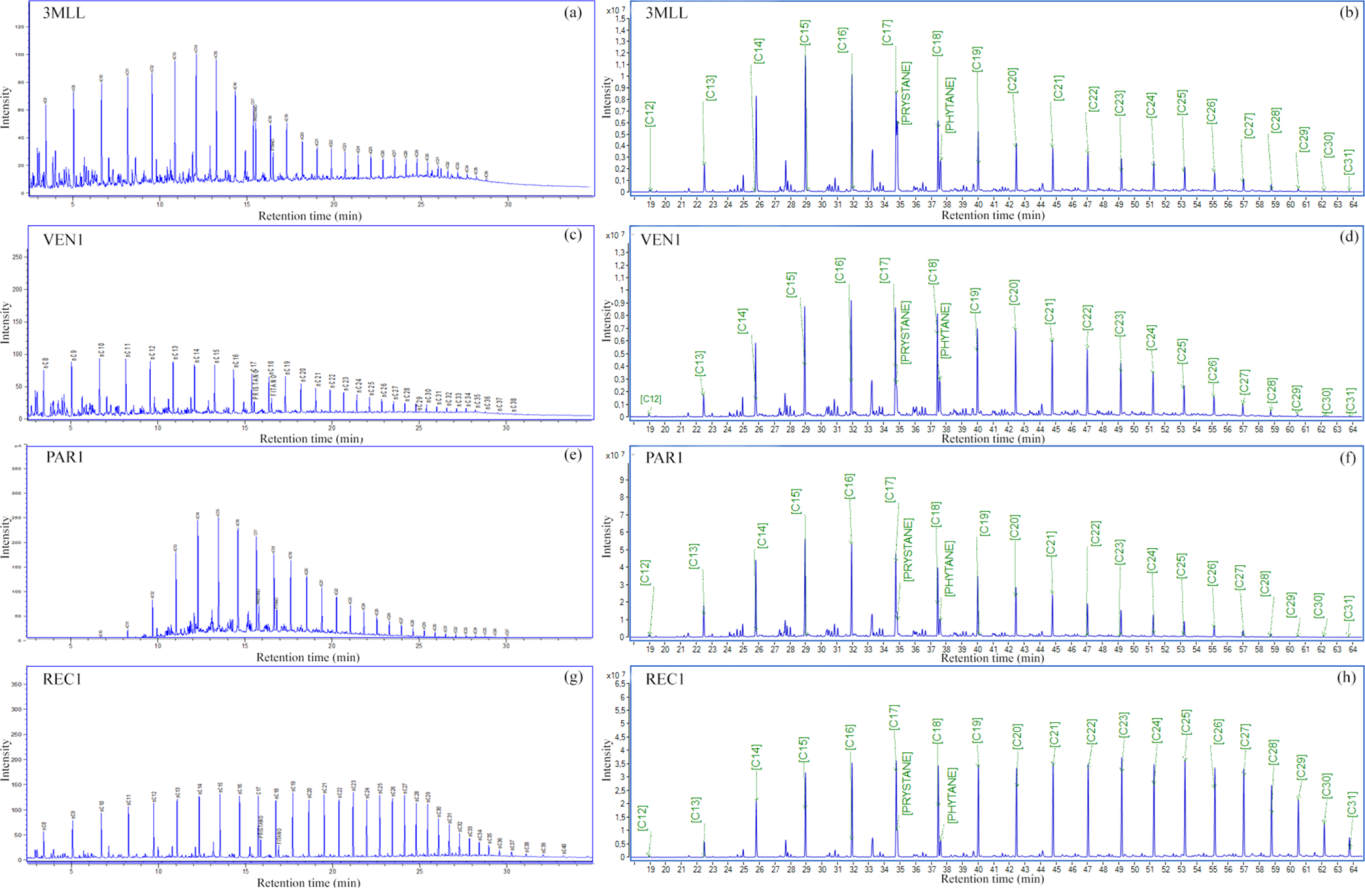
TPH chromatogram of the total oil by GC-FID (a–d) and GC-MS *m*/*z* 57 of the *n*-alkanes in the saturated fraction (e–h) of selected oils.

GC/FID chromatograms of selected samples are shown in Figure 4a–d (for the other samples, see Figures S1 and S2). All 14 samples analyzed showed profiles with no level of biodegradation. Selected geochemical ratios based on *n*-alkane and isoprenoid distributions are shown in Table 3. High Pr/*n*-C_17_ and Ph/*n*-C_18_ ratios, inconsistent with paleodepositional interpretations, indicate the biodegradation of oils.^35^ In Table 3, it is possible to observe the consistency in the values of all oil samples, and discrepant values are not verified. The absence of a complete series of 25-norhopanes (Table S4) in crude oils also indicates the nonbiodegradation of samples.^2,36^

**Table 3:** *n*-Alkanes, Pristane, and Phytane Parameters for the Oil Samples^a^

ratio	REC 1	REC 2	POT 1	POT 2	3 MLL	6 CHT	9 LL	SERG	PAR 1	PAR 2	COL 2	COL 1	VEN 1	VEN 2
Pr/Ph	0.29	0.62	0.40	0.43	1.20	0.86	0.85	0.52	0.39	0.72	0.43	1.11	0.12	0.09
CPI	1.25	1.23	1.19	1.18	0.95	1.08	1.07	1.20	0.94	1.16	0.90	0.97	0.86	1.00
Pr/C_17_	0.13	0.13	0.55	0.26	0.71	0.80	0.57	0.19	0.08	0.09	0.34	0.30	0.084	0.08
Ph/C_18_	0.24	0.24	0.65	0.52	0.59	0.71	0.56	0.29	0.33	0.11	0.40	0.31	0.45	0.51

a Pr/Ph= pristane/phytane; CPI= carbon preferential index; Pr/C_17_= pristane/*n*-C_17_; Ph/C_18_ = phytane/*n*-C_18_

N-markers are another class of compounds employed to evaluate biodegradation. The increase of CA and BCA concentrations in oils indicates higher biodegradation levels (Table S5). The high concentrations of CA and BCA observed in samples VEN 1 and 2, COL 1 and 2, PAR 1 and 2 and lower values in samples REC 1, REC 2, POT 1, and POT 2 (Table S5) suggest that these compounds had concentrations influenced by the source of organic matter or the conditions of the depositional paleoenvironment of their source rock.

Previous studies^37,38^ with classical biomarkers in crude oil indicate that low UCM and good preservation of *n*-alkanes are fundamental to determining biodegradation. Thus, in accordance with the results, the oil samples evaluated are nonbiodegraded.

**Figure 5:**
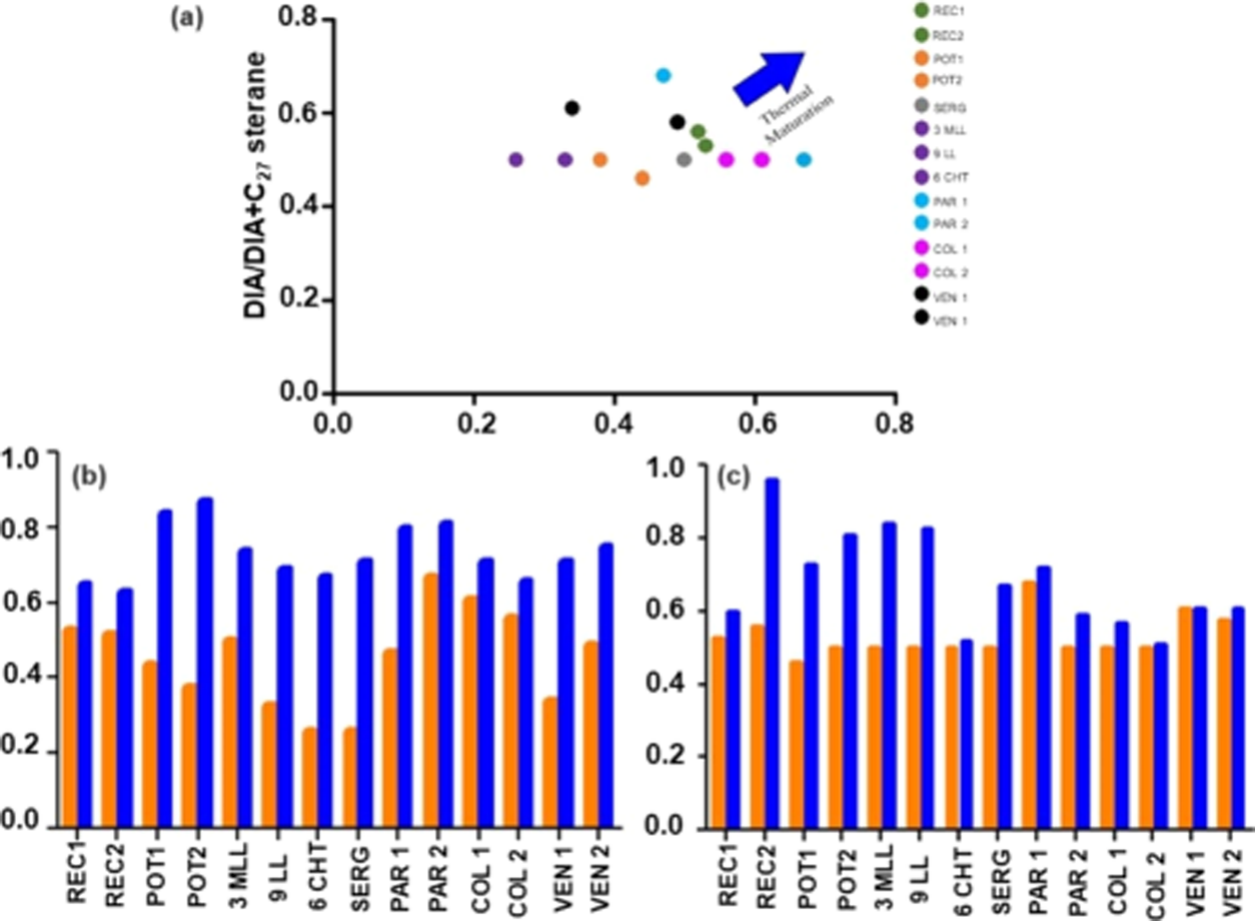
Evaluation of the thermal maturity for oil samples based on DIA/DIA+C_27_ e TS/TS+Tm (a), 4MCA/4MCA+CA DIA/DIA+C_27_ (b), and 1MCA/1MCA+3MCA DIA/DIA+C_27_ (c).

The thermal maturation was evaluated by the ratio of the biomarkers diasteranes/steranes C_27_ versus Ts/(Ts + Tm) and by ratios of N-markers methylcarbazoles.^2,13^ According to Figure 5a, a minimal increase in the values of DIA/DIA+C27 and Ts/(Ts+Tm) was observed. Thus, there is no clear trend that suggests maturity only by these biomarkers. When methylcarbazole ratios (4 MCA/4 MCA + CA and 1 MCA/1 MCA + 3 MCA) were employed, the values varied from 0.5 to 1.0 for all samples (Table S7), indicating maturity. A new correlation was proposed using biomarkers DIA/DIA+ STERANE C_27_ with N-markers 4 MC/4 MC + CA and 1 MC/1MC + 3 MC (Figure 5b,c). A minimum variation was observed for all samples, similar to the previous ones, confirming maturity.

The carbon preference index (CPI) is employed to evaluate the thermal maturity of petroleum by the relative abundance of odd versus even carbon numbers of *n*-alkanes.^4,53^ Values of 1.0 indicate a mature oil. Values < 1.0 are uncommon and typify low-maturity oils.^2^ In Table 3, it is possible to observe that for all samples the CPI values were around 1, thus, mature oil.

As more *n*-alkanes are generated from kerogen by cracking, the Pr/C_17_ and Ph/C_18_ parameters tend to decrease with thermal maturity.^53,57^ The results for the Pr/C_17_ and Ph/C_18_ ratios (Table 3) presented values < 1 for all samples, some lower than 0.1. Thus, these parameters also confirm the thermal maturity of all samples.

#### 3.2.2 Origin and Input of Organic Matter

The association of compounds such as cholesterol and cholestane; fitol and pristane, and phytane present in living organisms allows infer the type of organism that contributed to the formation of kerogen during the diagenesis of organic matter.^2^

Oils generated by the organic matter of continental and marine origins can be differentiated by *n*-alkanes, with predominance of higher molecular mass (C_25_ to C_33_) for continental origins and of lower molecular mass (C_15_ to C_33_), in marine origin.^39^ Samples REC1, REC2, POT 1, POT 2, and SERG present a greater abundance of low- to high-molecular-weight *n*-alkanes (mainly from C_13_ to C_25_), confirming that oils were generated by organic matter from freshwater lakes. On the other hand, in samples COL 1, COL 2, VEN 1, VEN 2, PAR 1, PAR 2, 3 MLL, 9, LL, and 6 CHT, there is a predominance of low-molecular-weight *n*-alkanes from C_9_ to C_16_, indicating organic matter deposited in a salinity context (Figure 4e–h).

Samples REC1, REC2, POT 1, POT 2, and SERG present a greater abundance of low- to high-molecular-weight *n*-alkanes (mainly from C_13_ to C_25_), confirming that oils were generated by organic matter from freshwater lakes. On the other hand, in samples COL 1, COL 2, VEN 1, VEN 2, PAR 1, PAR 2, 3 MLL, 9, LL, and 6 CHT, there is a predominance of low-molecular-weight *n*-alkanes from C_9_ to C_16_, indicating organic matter deposited in a salinity context (Figure 4f–h).^40^ In restricted depositional environments generally, there is no input from terrestrial plants, providing low hydrodynamics, high algal productivity, and high relative salinity (due to evaporation).

**Figure 6:**
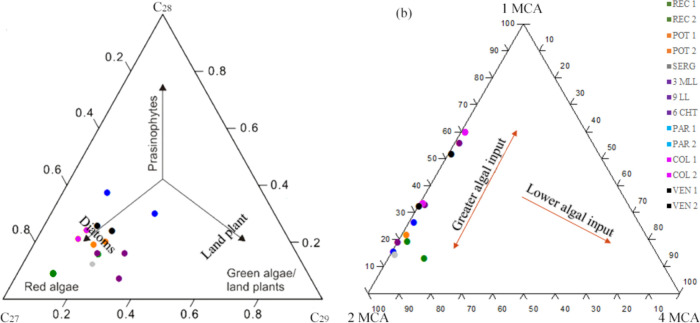
Ternary diagrams employed for organic matter inputs evaluation. Distribution of regular steranes C_27_, C_28_, and C_29_ (a) and isomeric distribution of methylcarbazoles (b).

The proportion between the regular steranes C_27_, C_28_, and C_29_ reflects the type of organic matter in the depositional environment.^2^ C_27_ steranes are characteristic of phytoplankton (algae), C_28_ steranes of fungi, plankton, and algae, and C_29_ steranes of higher plants.^41,42^ In the ternary diagram (Figure 6a), the predominance of C_27_ steranes in REC1, REC2, POT 1, POT 2, and SERG samples suggests a different origin than would be expected for samples of freshwater lacustrine origin, i.e., the greater amount of C_28_. The higher proportion of C_27_ and C_29_ steranes in oil samples generated by freshwater lacustrine organic matter from several Brazilian sedimentary basins^43^ confirms the interpretation of REC1, REC2, POT 1, POT 2, and SERG samples. These environments receive large contributions from higher plant material, whose precursor sterols are mainly C_29_, while C_27_ is derived from lacustrine phytoplankton. A previous study in the Recôncavo Basin (REC) also observed the predominance of C_27_ sterane, even though the samples were derived from organic material of freshwater lacustrine origin.

The higher proportion of C_27_ sterane justifies the abundance of tricyclic terpanes in relation to hopanes (tricyclic/hopane ratio) for the samples studied (Table S6). The tricyclics/hopanes ratio is a parameter indicative of organic matter input based on the ratio between the specific biological markers of tricyclics (originate from algal organic matter) and hopanes (originate from bacteria).^2^

The abundance of C_27_ sterane and the greater proportion of tricyclics in relation to hopanes are closely linked to the ternary diagram of the MCA of the N-markers. Samples with high levels of alginite (maceral algae, whether lacustrine or marine) present higher proportions of 1-MCA and 2-MCA than 4-MCA.^13^ Thus, the ternary diagram of these N-markers of the samples (Figure 6b) indicates an entry of organic material of the algae type.

**Figure 7:**
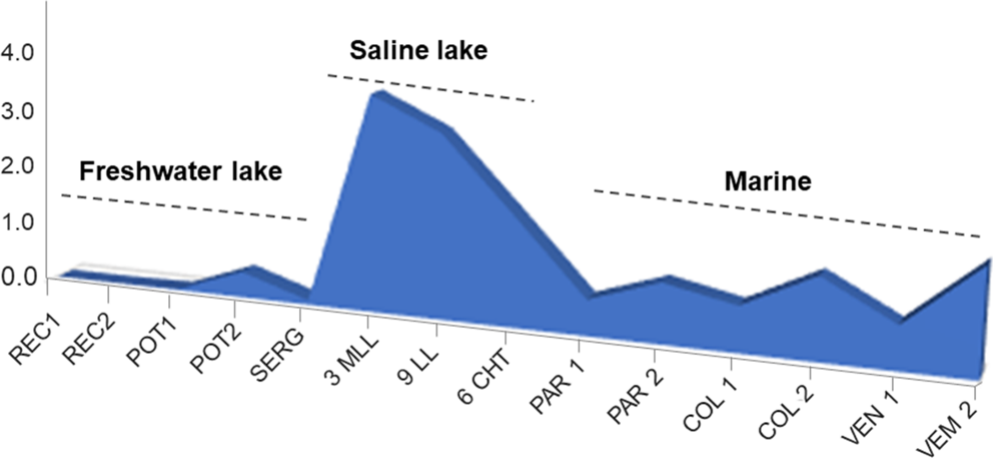
Concentration of carbazoles (CA) in the oil samples in the study.

**Figure 8:**
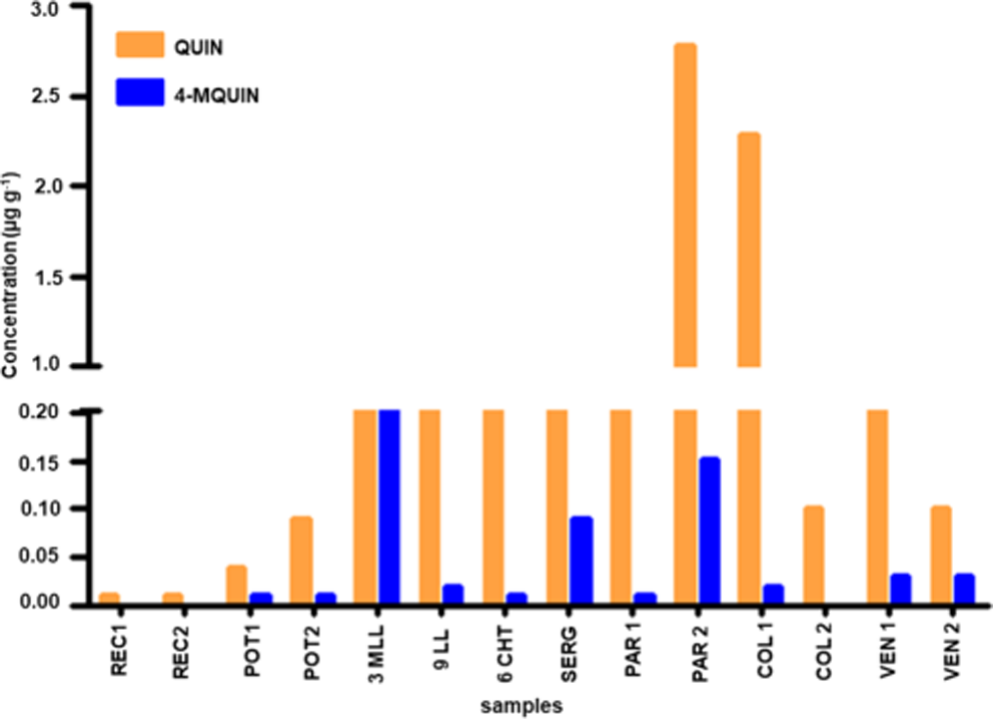
Distribution of quinoline (QUIN) and 4-methyl quinoline (4-MQUIN) concentrations in the oil samples.

Low concentrations of N-markers were found in nonmarine samples of petroleum source rocks.^44^ In the present study, a low concentration of CA in oil samples was generated by lacustrine organic matter (REC1, REC2, POT 1, POT 2, and SERG) (Figure 7). For the basic N-markers, low concentrations of quinoline and proportionally low 4-methyl quinoline concentrations were also observed in the same samples (Figure 8).

**Figure 9:**
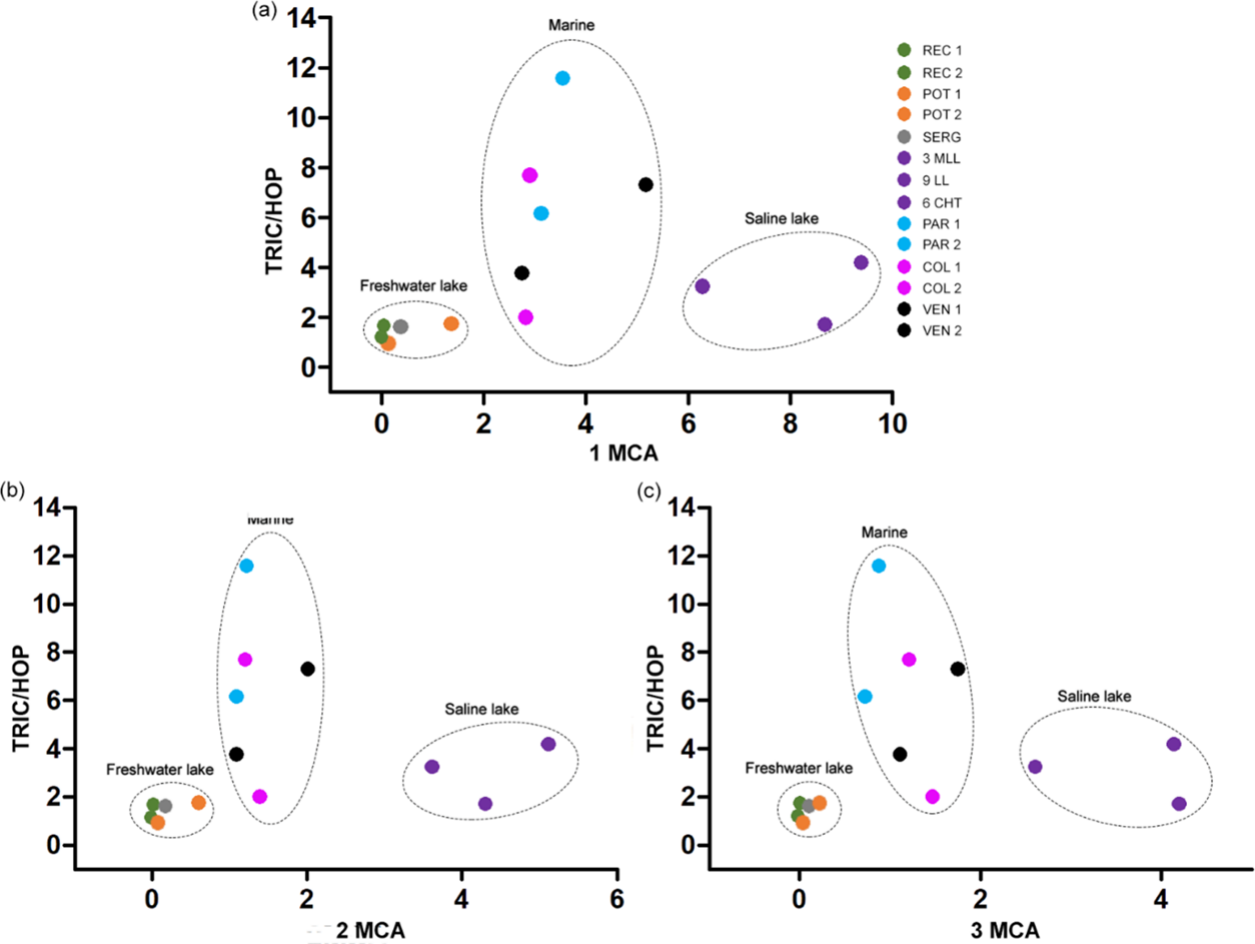
Correlation of 1-methylcarbazole (1-MCA) (a), 2-methylcarbazole (1-MCA) (b), and 3-methylcarbazole (1-MCA) (c) with tricycles/hopanes (TRIC/HOP).

Carbazole isomers, mainly 1-MCA were observed in hydrocarbon source rocks deposited in a marine context.^9,10,13^ The comparison between 1 MCA, 2 MCA, and 3 MCA concentrations and the TRIC/HOP ratio facilitated differentiation of the depositional environments of samples in the study (Figure 9). It is possible to identify the three types of environments examined here, fresh lake (REC 1, REC 2, POT 1, POT 2, and SERG), marine (PAR 1, PAR 2, COL 1, COL 2, VEN 1, and VEN 2) and saline lacustrine (3 MLL, 6CHT, and 9LL). These results demonstrated that the main separation factor for the oil samples was probably the salinity.

**Figure 10:**
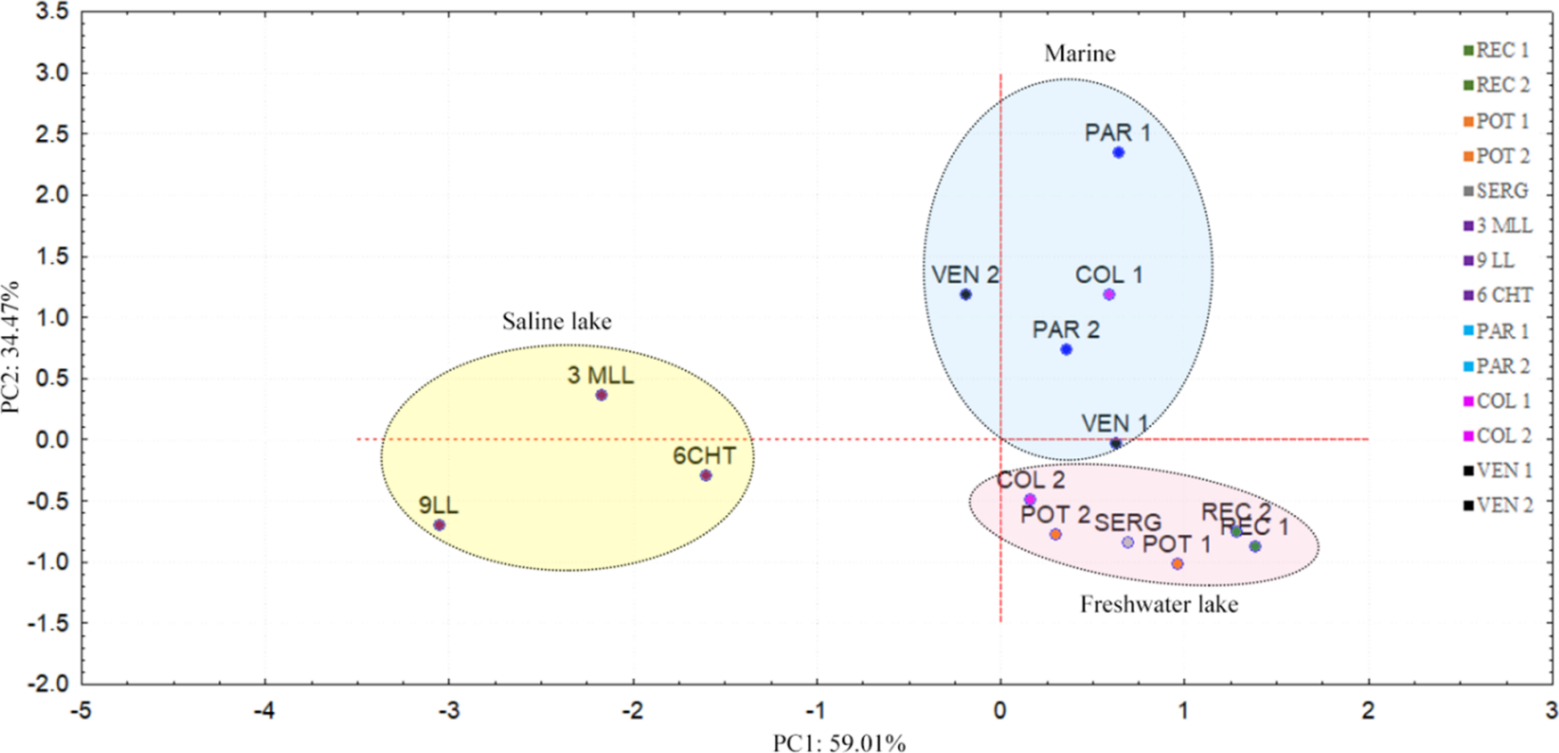
Principal component analysis showing the grouping of samples.

The application of PCA to evaluate the correlation between CA compounds and tricyclic/hopane ratio (Figure 10) showed a good grouping of samples according to their depositional environments. Samples derived from organic material of marine origin in the positive quadrant were mainly favored by TRIC/HOP (PAR 1, PAR 2, COL 1, VEN 1, and VEN 2). Samples 3MLL, 9LL, and 6CHT, from organic matter of saline lacustrine origin, were favored by the weights of carbazoles and benzocarbazoles.^26,45^ The other samples derived from organic material of fresh lake origin (REC 1, REC 2, POT 1, POT 2, and SERG) except COL 2, derived from organic material of marine origin, had a greater contribution from TRIC/HOP.

**Figure 11:**
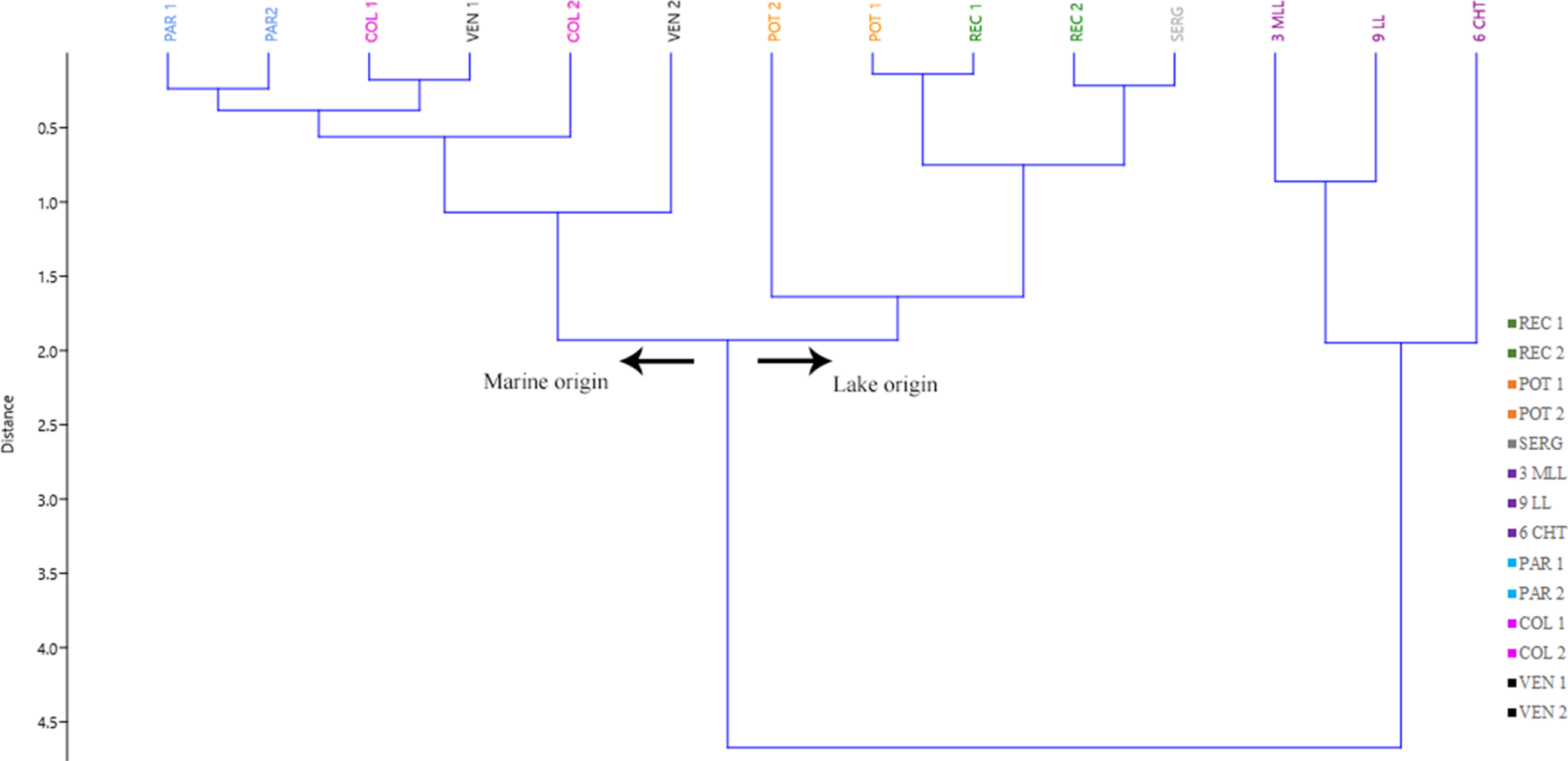
Cluster analysis for crude oil samples based on C_27_ sterane and methylcarbazoles (1-, 2-, and 3-methylcarbazole).

The HCA (Figure 11) grouped samples based on the similarities between the oils. From C_27_ steranes, a biological marker, to methylcarbazoles (1-MCA, 2MCA and 3MCA), N-markers. It was possible to establish that the input of organic matter was the same for all samples and that the main factor that caused the groupings was the depositional environment. Though the input is similar for the samples (algal input, as shown in Figure 6), there is a distinction in the composition of constituents due to the difference in the N-markers. This result indicates control of the depositional environment in the composition of the elements present in the algae.

**Figure 12:**
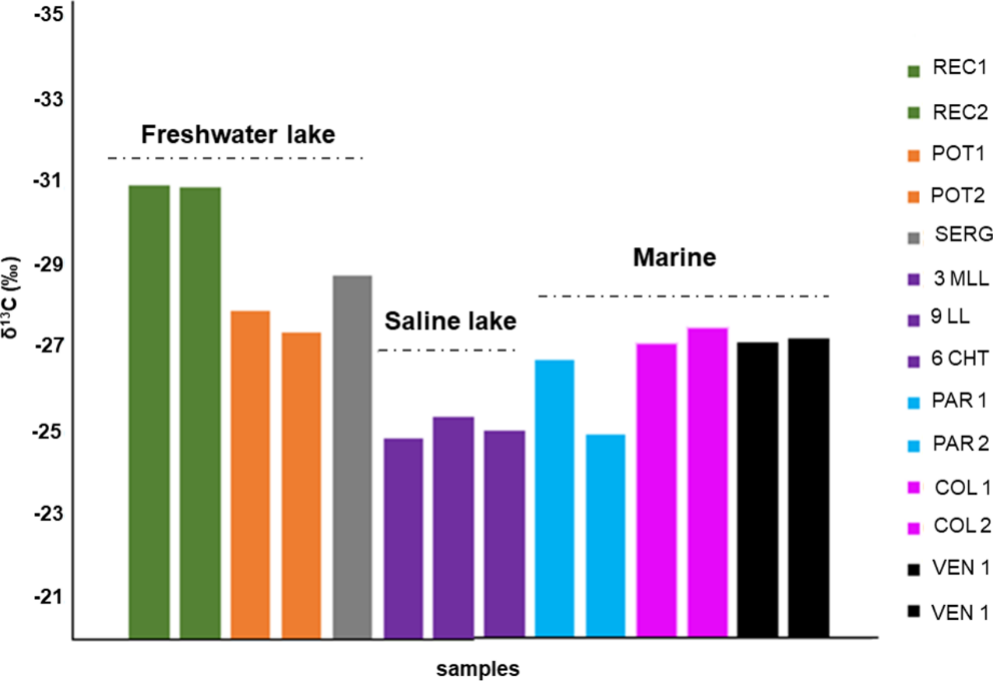
Organic carbon isotope δ^13^C distribution for the samples.

Organic stable carbon isotope analysis was also used to assess the origin of OM.^17,46,47^ In the samples, it was observed that oils from marine organic matter have proportionally more positive δ^13^C values when compared to those of lacustrine organic matter (REC 1 and 2, POT 1 and 2, SERG) (Figure 12).

Previous studies in oils from different regions, including the Brazilian marginal basins (Reconcavo, Potiguar, Sergipe, Santos, and Campos), reported that organic matter present in freshwater lake environments is generally enriched in ^12^C, recorded with isotopic values lower than −28‰.^43,48^ This result is in accordance with the values of REC 1 and 2 samples, POT 1 and 2, and SERG (Figure 12). On the other hand, in saline environments, due to relative enrichment in ^13^C, δ13C values were higher than −28‰, similar to what was observed for samples 3MLL, 6CHT, 9LL, PAR 1, PAR 2, COL 1, COL 2, VEN 1, and VEN 2. This behavior is due to density; saline environments do not have the same ease of exchanging CO_2_ with the atmosphere as freshwater environments, making it more enriched in CO_2_ containing more ^13^C.^49^

For oils of freshwater lake origin, isotopic values lower than −28‰ were obtained in POT 1, POT 2, REC 1, REC 2, and SERG samples. Similar behavior, with more negative values correlated with the respective source rocks paleoenvironments, was found in the Potiguar Basin.^50^

Previous studies have employed the δ^13^C parameter to classify and compare different oil groups according to origins as stratified environment, freshwater lacustrine, freshwater transitional lacustrine, and relatively closed saline lacustrine.^51^ Thus, this indicates that isotopic composition, such as in the present study, can very well demarcate differences in depositional environments.

#### 3.2.3 Depositional Conditions

The Pr/Ph ratio is used as a parameter for redox conditions. Organic matter deposited under anoxic conditions presents values < 0.8, while values > 3.0 are associated with organic matter deposited under more oxic conditions. Values between the two ranges (0.8 and 3.0) suggest intermediate (dysoxic) deposition conditions.^2,52^ Pr/Ph ratio determines the deposition conditions and helps in the interpretation of the depositional paleoenvironment of petroleum source rocks. For example, in the depositional paleoenvironment of petroleum source rocks of lacustrine origin from the Cretaceous, a high Pr/Ph ratio in the organic matter deposition environment occurred under an oxidizing context.^53^

**Figure 13:**
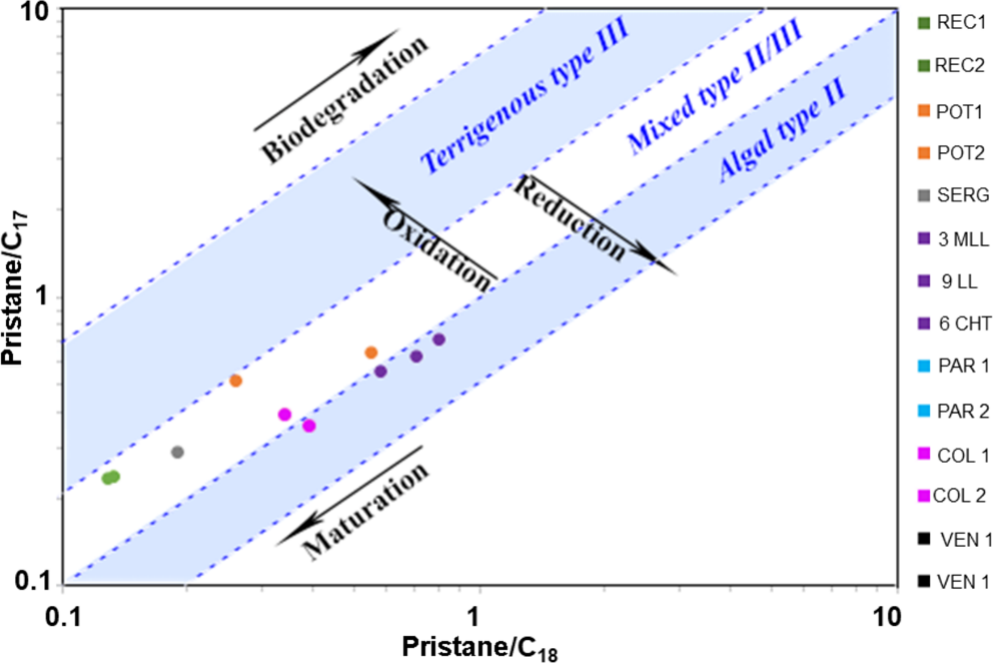
Pristane/C_17_ and phytane/C_18_ ratios for crude oils indicate the conditions of the depositional paleoenvironment. The samples in the graph are those whose values are superior to the axis.

The Pr/Ph values in Table 3 indicate that the organic matter that originated in most samples was deposited under anoxic conditions (POT 1, POT 2, REC 1, REC 2, SERG, 9 LL, 6 CHT, PAR 1, PAR 2, COL 2, VEN 1, and VEN 2). The origin of MLL and COL 1 was similar to dysoxic conditions (3 MLL and COL 1). The results for VEN 1 and VEN2 are in accordance with previous studies in oils from the Maracaibo Basin, Venezuela, where Pr/Ph < 0.8 indicated anoxic conditions. The relationship between Pr/C_17_ and Ph/C_18_ is also used to evaluate depositional conditions.^2^ In Figure 13, it is possible to confirm the data in Table 3, where the majority of samples were formed in anoxic context and some in more dissoxic conditions.

**Figure 14:**
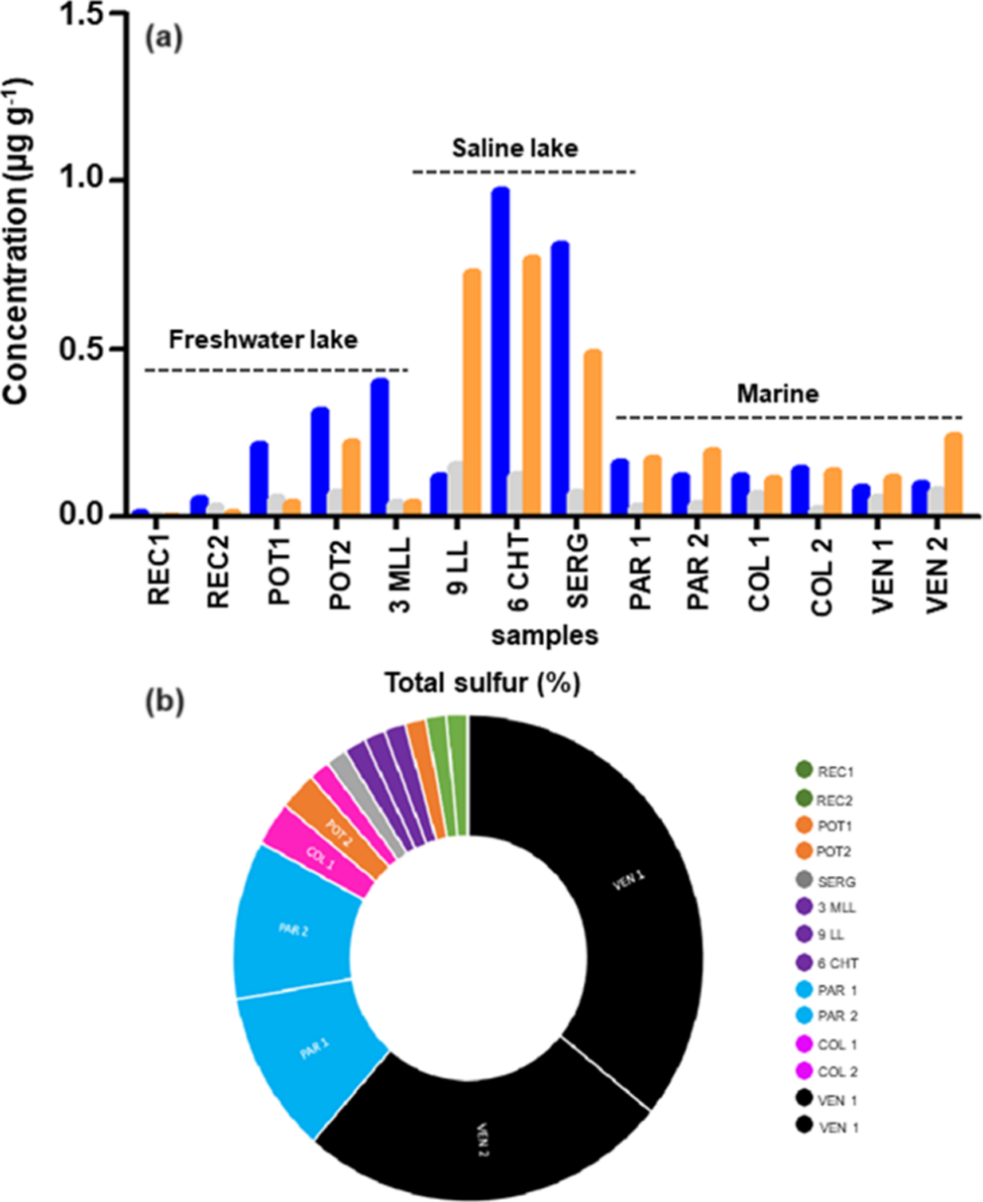
Concentrations of benzocarbazoles (a) and total sulfur values (b) for petroleum samples.

The type of kerogen and its precursor organic matter for each sample is shown in Figure 14a, and all samples fit in types II and III. Escobar et al.^37^ using this same parameter, also classified the precursor organic matter of the Venezuelan oils under study as type II. The REC 1 and REC 2 oils originate from organic matter type II and III (Figure 14a) and are compatible with previous studies of oils from the Recôncavo Basin,^54,55^ indicating oxidizing conditions of the depositional environment.

Another parameter to evaluate deposition conditions is the N-markers from the benzocarbazoles class.^56^ More reducing conditions present low values for the benzocarbazoles associated with high sulfur values, while high values are related to more oxidizing conditions and low values of sulfur.

In oils originating from marine organic matter, benzocarbazoles are more intense in environments with higher salinity conditions.^9,10^ In samples, the benzocarbazoles isomers (Figure 13a) were different for saline lacustrine (3MLL, 6CHT, and 9LL) > marine (PAR 1, PAR 2, COL 1, COL 2, VEN 1, and VEN 2) > fresh lacustrine samples (REC1, REC 2, POT 1, POT 2, and SERG). On the other hand, the highest sulfur values were not directly related to the highest concentrations of benzocarbazoles (Figure 14a). Therefore, for the samples under study, the main factor controlling the amounts of benzocarbazoles was the salinity of the depositional paleoenvironment of organic matter.

Oils with higher sulfur contents are associated with marine source rocks with low clay contents (carbonate and anhydrite) deposited under reducing conditions. On the other hand, oils with low sulfur concentrations are derived from siliciclastic source rocks.^29^ Thus, the VEN 1, VEN 2, COl 1, COl 2, PAR 1, and PAR 2 can be derived from source rocks with greater carbonate contributions (>sulfur content). The REC 1, REC 2, POT 1, POT 2, and SERG samples can be derived from source rocks with greater siliciclastic contributions (>sulfur content proportionally) (Figure 14b).

## 4 Conclusions

A chromatographic method optimization employing standards, SRM, and Kóvats IR allowed the reliable identification and quantification of biomarkers and N-markers for posterior geochemistry applications. The distribution of *n*-alkanes, saturated biomarkers, nitrogen markers and δ^13^C values indicated that the depositional paleoenvironment of the source rocks was freshwater lake source rocks (REC1, REC 2, POT 1, POT 2, and SERG), marine paleoenvironment (VEN 1, VEN 2, COl 1, COl 2, PAR 1, and PAR 2) and saline lake (3MLL, 6CHT, 9LL). The predominance of tricycles in relation to hopanes and the greater proportion of 1-MCA and 2-MCA isomers indicated the contribution of algal organic matter to all source rocks in the studied samples. The ratio of Pr/Ph isoprenoids, the distribution of benzocarbazoles, and the low sulfur values allowed indicated that the precursor organic matter of the samples was predominantly deposited in the context of anoxia.

Based on all parameters presented, it was possible to assess the depositional paleoenvironment with different petroleum samples. The integration of all parameters analyzed in this research allowed us to refine the interpretations and differentiate the oils under study according to their respective depositional environments.

## Supporting Information Available

*n*-Alkanes, isoprenoids, N-markers identification, retention time (*t*_R_), and concentrations in the crude oil samples (Tables S1–S5); diagnostic ratios and concentration of saturated and N-markers in crude oil samples (Tables S6–S7); and TPH chromatogram of total oil GC/FID and GC-MS chromatogram of the saturated fractions of oils (Figures S1 and S2) (PDF)
