# Supplementary material for: An Integrated Assessment of Different Depositional Paleoenvironment Using Nitrogen Markers and Biomarkers after Chromatographic Methods Optimization
Source: ACS Omega. 2024 Sep 5;9(37):38633–47. doi: 10.1021/acsomega.4c04189 (PMC11411675; doi:10.1021/acsomega.4c04189)
Supplement: Supplementary file 1 — ao4c04189_si_001.pdf [file ao4c04189_si_001.pdf]

SUPPLEMENTARY MATERIAL

An integrated assessment of different depositional paleoenvironment using nitrogen markers and biomarkers after chromatographic methods optimization

Flávia Lima e Cima Miranda [a], Diego Nery do Amaral [a,d], José Roberto Cerqueira [a], Karina Santos Garcia [a], Antônio Fernando de Souza Queiroz [a], Maria Elisabete Machado [a,b,c*]

[a] Programa de Pós-Graduação em Petróleo e Meio Ambiente, Instituto de Geociências, Universidade Federal da Bahia, Salvador, BA, Brazil

[b] Departamento de Química Analítica, Instituto de Química, Universidade Federal da Bahia, Salvador, BA, Brazil

[c] Centro Interdisciplinar de Energia e Ambiente, Universidade Federal da Bahia, Salvador, BA, Brazil

[d] Departamento de Ciências Exatas, Universidade Estadual de Feira de Santana, BA, Brazil

*Correspondent author: maria.elisabete@ufba.br

ORCID: 0000-0001-8289-4842

Table S1. *n*-alkanes and isoprenoids identification and retention time (t_R) based on standards

n°	Compound	Abbreviation	t_R (min)
1	*n*-Nonane	C_9	8.70
2	*n*-Decane	C_{10}	11.61
3	*n*-Undecane	C_{11}	15.31
4	*n*-Dodecane	C_{12}	18.96
5	*n*-Tridecane	C_{13}	22.46
6	*n*-Tetradecane	C_{14}	25.79
7	*n*-Pentadecane	C_{15}	28.93
8	*n*-Hexadecane	C_{16}	31.91
9	*n*-Heptadecane	C_{17}	34.74
10	Hexadecane d_{34}	HEX-d_{34}	31.19
11	*n*-Octadecane	C_{18}	37.43
12	*n*-Nonadecane	C_{19}	39.99
13	*n*-Eicosane	C_{20}	42.44
14	*n*-Heneicosane	C_{21}	44.78
15	*n*-Docosane	C_{22}	44.02
16	*n*-Tricosane	C_{23}	49.18
17	*n*-Tetracosane	C_{24}	51.24
18	*n*-Pentacosane	C_{25}	53.29
19	Eicosane d_{42}	EIC-d_{42}	41.65
20	*n*-Hexacosane	C_{26}	55.13
21	*n*-Heptacosane	C_{27}	56.98
22	*n*-Octacosane	C_{28}	58.76
23	*n*-Nonacosane	C_{29}	60.48
24	*n*-Triacontane	C_{30}	62.14
25	*n*-Hentriacontane	C_{31}	63.75
26	*n*-Dotriacontane	C_{32}	65.31
27	Ocatacosne d_{58}	OCT-d_{58}	57.89
28	Pristane	Pr	34.79
29	Phytane	Ph	37.58

Table S2. Quantification of *n*-alkane compounds in the crude oil samples

Compound	REC 1	REC 2	POT 1	POT 2	3 MLL	6CHT	9LL	SERG	PAR 1	PAR 2	COL 1	COL 2	VEN 1	VEN 2
	Concentration (mg g^{-1})													
C_9	n.d.	n.d.	n.d.	n.d.	n.d.	n.d.	n.d.	n.d.	n.d.	0.01	n.d.	n.d.	n.d.	n.d.
C_{10}	0.01	0.01	0.05	n.d.	n.d.	0.02	0.07	n.d.	0.12	0.10	0.03	0.02	n.d.	0.02
C_{11}	0.06	0.07	0.07	0.11	0.02	0.07	0.11	0.07	0.18	0.10	0.03	0.05	0.02	0.02
C_{12}	0.39	1.50	0.75	1.10	0.35	1.40	0.82	0.53	3.90	3.80	0.40	2.30	0.31	0.36
C_{13}	5.20	18.2	5.10	6.10	3.90	12.4	4.20	2.50	38.6	34.1	3.40	11.4	2.80	1.80
C_{14}	18.4	45.7	15.9	16.6	13.2	30.6	16.6	11.1	93.2	65.9	9.40	20.6	9.50	7.90
C_{15}	26.7	58.6	25.9	26.7	17.5	37.9	26.5	23.1	113.9	75.5	13.2	23.6	12.5	14.5
C_{16}	44.6	43.2	2.50	23.0	11.3	28.6	22.3	28.0	57.6	65.8	21.0	16.0	10.3	22.9
C_{17}	23.3	48.7	19.1	21.8	6.80	15.3	14.9	25.6	73.9	51.8	9.40	14.8	9.60	12.8
C_{18}	43.4	42.4	40.8	25.6	6.70	19.7	18.0	31.5	43.8	55.6	18.9	14.4	8.90	21.2
C_{19}	44.9	44.1	40.9	25.9	5.50	17.4	16.6	31.9	36.4	54.7	17.8	13.7	7.90	19.3
C_{20}	44.3	44.4	40.5	25.7	4.40	14.6	14.8	31.2	30.9	52.1	16.4	13.3	7.60	18.4
C_{21}	47.6	48.6	41.4	26.8	3.70	14.1	14.3	33.4	26.3	51.7	14.8	13.0	6.90	17.2
C_{22}	46.7	49.3	40.0	25.9	3.40	13.4	15.0	32.4	21.5	49.5	13.5	12.7	6.10	16.3
C_{23}	51.5	53.6	41.9	27.4	3.20	12.9	14.7	34.5	16.5	48.3	10.9	12.1	5.00	13.9
C_{24}	47.6	48.7	38.7	24.9	2.70	12.0	14.5	33.1	13.2	43.0	8.30	11.2	4.00	12.4
C_{25}	50.2	50.2	36.6	23.8	2.20	11.2	13.0	35.4	9.60	40.7	5.40	10.1	2.80	9.40
C_{26}	24.0	22.7	13.7	11.6	2.60	7.20	7.40	23.5	6.60	16.9	8.80	13.7	3.80	5.60
C_{27}	25.2	22.2	12.2	10.7	1.80	6.00	6.30	25.6	3.80	14.5	4.80	10.7	2.20	4.10
C_{28}	19.5	15.3	8.30	7.60	1.10	4.90	4.80	20.4	2.00	9.80	0.20	6.20	1.20	2.70
C_{29}	16.5	11.0	5.40	5.10	0.40	3.00	2.70	16.2	0.80	6.30	0.70	3.40	0.40	1.50
C_{30}	9.0	5.00	2.40	2.30	0.20	1.50	1.20	7.90	0.30	3.00	n.d.	1.20	0.10	0.80
C_{31}	5.30	2.20	0.90	1.20	n.d.	0.80	0.40	3.70	0.10	1.20	n.d.	0.40	n.d.	0.3

C_{32}	2.00	0.70	0.30	0.30	n.d.	0.20	0.20	1.00	nd	0.40	n.d.	n.d.	n.d.	0.10
Pr	3.10	6.30	10.5	5.70	4.80	12.2	8.60	4.80	5.60	4.60	3.20	5.80	0.80	1.10
Ph	10.5	10.1	26.4	13.3	4.0	14.1	10.1	9.2	14.4	6.4	7.5	5.2	4.4	10.8

Table S3. Identification of Hopanes and steranes biomarkers

n°	**Compound**	**Abreviation**	**t_R (min)**	**m/z**	**Identification**	**Reference**
1	C19 tricyclic terpane	TR19	42.42	191	elution order	Rangel et al, 2017
2	C20 tricyclic terpane	TR20	42.85	191	retention index	Yoneyam, 1996
3	C21 tricyclic terpane	TR21	43.10	191	elution order	Rangel et al, 2017
4	C22 tricyclic terpane	TR22	43.42	191	elution order	Rangel et al, 2017
5	C23 tricyclic terpane	TR23	44.14	191	elution order	Rangel et al, 2017
6	C24 tricyclic terpane	TR24	44.34	191	elution order	Rangel et al, 2017
7	C25tricyclic terpane[(a)]	TR25A	45.11	191	retention index	Yoneyam, 1996
8	C25tricyclic terpane[(b)]	TR25B	45.11	191	retention index	Yoneyam, 1996
9	C24 tetracyclic terpane	TET24	46.91	191	elution order	Rangel et al, 2017
10	C26 tricyclic terpane (a)	TR26A	49.02	191	elution order	Rangel et al, 2017
11	C26 tricyclic terpane (b)	TR26B	50.13	191	elution order	Rangel et al, 2017
12	C28 tricyclic terpane (a)	TR28A	54.05	191	elution order	Rangel et al, 2017
13	C28 tricyclic terpane (b)	TR28B	54.17	191	elution order	Rangel et al, 2017
14	C29 tricyclic terpane (a)	TR29A	57.74	191	elution order	Rangel et al, 2017
15	C29 tricyclic terpane b)	TR29B	57.96	191	elution order	Rangel et al, 2017
16	18α(H),21 β(H)-22,29 ,30-trisnorhopane	Ts	59.82	191	retention index	Hwang, 1990
17	17α(H),21β(H)-22,29,30-trisnorhopane	Tm	60.59	191	Standard	-
18	C30 tricyclic terpane 1	TR30A	60.73	191	elution order	Rangel et al, 2017
19	C30 tricyclic terpane2	TR30B	61.03	191	elution order	Rangel et al, 2017
20	17α(H),18α(H),21 β(H)-28,30-bisnorhopane	H28	62.18	191	elution order	Rangel et al, 2017
21	17α(H).21 β(H)-30-norhopane	H29	63.08	191	retention index	Yoneyam, 1996
22	17α(H-)diahopano	DH30	63.59	191	elution order	Eiserbeck et al, 2012
23	17α(H),21 β(H)-norhopane (moretane)	M29	63.71	191	elution order	Rangel et al, 2017
24	17α(H),21 β(H)-hopane	H30	64.10	191	Standard	-
25	17a(H)-30-nor-29-homohopane	NOR30H	64.42	191	elution order	Rangel et al, 2017
26	17 βH),21a(H)-hopane (moretane)	M30	64.76	191	elution order	Rangel et al, 2017
27	22S-17a(H),21 β(H)-30-homohopane	H31S	65.67	191	elution order	Rangel et al, 2017
28	22R -17 a(H),21 β(H)-30-homohopane	H31R	65.85	191	elution order	Rangel et al, 2017
29	Gammacerane	GAM	66.19	191	Standard	-
30	22S-17a(H),21 β(H)-30,31-bishomohopane	H32S	66.57	191	elution order	Rangel et al, 2017

31	22R-17a(H),21 β(H)-30,31-bishomohopane	H32R	67.16	191	elution order	Rangel et al, 2017
32	22S-17u(H),21 β(H)-30,31 ,32-trishomohopane	H33S	66.94	191	elution order	Rangel et al, 2017
33	22R-17u(H),21 β(H)-30,31 ,32-trishomohopane	H33R	67.21	191	elution order	Rangel et al, 2017
34	22S-17a(H),21β(H)-30,31 ,32,33-tetrakishomohopane	H34S	68.66	191	elution order	Rangel et al, 2017
35	22R-17a(H),21β(H)-30,31 ,32,33-tetrakishomohopane	H34R	69.08	191	elution order	Rangel et al, 2017
36	22S-17a(H),21β(H)-30,31 ,32,33,34-pentakishomohopane	H35S	70.78	191	elution order	Rangel et al, 2017
37	22R-17a(H),21β(H)-30,31 ,32,33,34-pentakishomohopane	H35R	71.44	191	elution order	Rangel et al, 2017
38	5β-Cholane	I.S	53.32	217	Standard	-
39	C21 5α(H), 14β(H), 17β(H)-sterane	S21	55.94	217	elution order	Rangel et al, 2017
40	C22 5α(H), 14β(H), 17β(H)-sterane	S22	56.51	217	elution order	Rangel et al, 2017
41	C27 20S-13β(H), 17a(H)-diasterane	DIA27S	58.69	217	elution order	Rangel et al, 2017
42	C27 20R -13β(H), 17a(H)-diasterane	DIA27R	58.82	217	elution order	Rangel et al, 2017
43	C27 20S-13α(H), 17β(H)-diasterane	DIA27SS	59.01	217	elution order	Rangel et al, 2017
44	C27 20R-13α(H), 17β(H)-diasterane	DIA27RR	59.36	217	elution order	Rangel et al, 2017
45	C27 20S-5α(H), 14a(H), 17a(H)-cholestane	C27S	61.69	217	retention index	Hwang, 1990
46	C27 20R -5α(H), 14β(H), 17~(H)-cholestane	C27BBR	61.89	217	retention index	Hwang, 1991
47	CZ7 20S-5α(H), 14β(H), 17β(H)-cholestane	C27BBS	62.07	217	retention index	Hwang, 1992
48	C27 20R-5a(H), 14a(H), 17a(H)-cholestane	C27R	62.25	217	retention index	Hwang, 1993
49	C28 20S-5α(H), 14a(H), 17a(H)-ergostane	C28S	62.58	217	retention index	Hwang, 1994
50	C28 20R-5α(H), 14β(H), 17β(H)-ergostane	C28BBR	62.76	217	retention index	Hwang, 1995
51	C28 20S-5a(H), 14β(H),17β(H)-ergostane	C28BBS	63.03	217	retention index	Hwang, 1996
52	C28 20R-5α(H), 14a(H), 17a(H)-ergostane	C28R	63.17	217	retention index	Hwang, 1997
53	C29 20S-5α(H), 14u(H), 17a(H)-stigrmastane	C29S	63.32	217	retention index	Hwang, 1998

54	C29 20R-5α(H), 14β(H), 17β(H)-stigmastane	C29BBR	63.43	217	retention index	Hwang, 1999
55	C29 20S-5α(H), 14β(H), 17β(H)-stigrmastane	C29BBS	63.76	217	retention index	Hwang, 2000
56	C2920R-5α(H), 14a(H), 17a(H)-stigrmastane	C29R	64.11	217	retention index	Hwang, 2001
57	poliprenóides tetracíclicos	TPP1	64.01	259	elution order	Rangel et al, 2017
58	poliprenóides tetracíclicos	TPP2	64.10	259	elution order	Rangel et al, 2017
59	Olean	olean	-	191	Standard	-

Table S4. Identified hopanes and steranes compounds and their respective concentrations in crude oil samples

Compound	REC 1	REC 2	POT 1	POT 2	3 MLL	6CHT	9LL	SERG	PAR 1	PAR 2	COL 1	COL 2	VEN 1	VEN 2
	Concentration (mg g^{-1})													
TR19	0.04	0.04	0.04	0.04	0.06	0.06	0.03	0.02	0.02	0.01	0.06	0.02	0.01	0.01
TR20	0.05	0.05	0.05	0.05	0.08	0.10	0.07	0.02	0.08	0.02	0.02	0.05	0.02	0.01
TR21	0.13	0.13	0.21	0.22	0.42	0.68	0.35	0.08	0.42	0.03	0.07	0.20	0.11	0.09
TR22	0.01	0.01	0.01	0.01	0.02	0.03	0.02	0.01	0.02	0.04	0.04	0.88	0.87	0.79
TR23	0.06	0.07	0.09	0.04	0.09	0.07	0.02	0.05	0.02	0.05	0.07	0.06	nd	0.23
TR24	0.02	0.02	0.04	0.03	0.07	0.01	0.12	0.01	0.11	0.18	0.05	0.02	0.14	0.01
TR25A	0.23	0.26	0.44	0.43	0.87	1.26	0.77	0.16	0.65	0.08	0.12	0.28	0.17	0.14
TR25B	n.d.	n.d.	n.d.	n.d.	n.d.	n.d.	n.d.	n.d.	n.d.	n.d.	n.d.	n.d.	n.d.	n.d.
TET24	0.05	0.05	0.05	0.05	0.11	0.19	0.10	0.01	0.10	0.01	0.02	0.03	0.09	0.05
TR26A	0.29	0.31	0.52	0.51	0.87	1.39	1.02	0.17	0.57	0.07	0.18	0.26	0.56	0.41
TR26B	0.22	0.23	0.38	0.39	0.60	0.95	0.63	0.12	0.37	0.05	0.15	0.17	0.24	0.23
TR28A	0.19	0.21	0.26	0.32	0.28	0.59	0.40	0.07	0.21	0.03	0.10	0.05	0.09	0.11
TR28B	0.12	0.13	0.17	0.16	0.15	0.33	0.24	0.04	0.09	0.02	0.05	0.02	0.04	0.06
TR29A	0.13	0.12	0.14	0.15	0.68	0.24	0.21	0.03	0.05	0.020	0.05	0.01	0.06	0.05
TR29B	0.13	0.11	0.14	0.14	0.08	0.23	0.20	0.03	0.02	0.02	0.03	0.01	0.06	0.05
Ts	0.25	0.20	0.26	0.37	0.10	0.20	0.14	0.06	0.06	0.02	0.06	0.02	0.04	0.03
Tm	0.22	0.19	0.33	0.61	0.05	0.58	0.39	0.06	0.07	0.01	0.04	0.01	0.07	0.03
TR30A	0.08	0.06	0.10	0.08	0.02	0.10	0.08	0.02	0.01	0.01	0.02	0.01	0.03	0.03
TR30B	0.10	0.00	0.09	0.13	0.04	0.16	0.12	0.01	0.02	0.01	0.02	0.01	0.05	0.04
H28	0.07	0.05	0.05	0.18	0.02	0.06	0.17	0.05	0.01	0.06	0.09	nd	0.02	0.01
H29	0.12	0.08	0.09	0.10	0.02	0.05	0.05	0.03	0.01	0.02	0.02	0.01	nd	nd
C29Ts	nd	nd	nd	nd	nd	nd	nd	nd	nd	nd	nd	nd	nd	nd
DH30	0.13	0.08	0.08	0.13	0.02	0.11	0.05	0.02	0.01	0.01	0.11	nd	0.01	0.01

M29	0.02	0.01	0.02	0.03	0.01	0.02	0.02	0.03	nd	0.01	0.03	nd	nd	nd
H30	1.49	0.10	1.26	2.30	0.45	0.16	1.13	0.23	0.11	0.02	0.13	0.05	0.14	0.06
NOR30H	0.05	0.04	0.05	0.09	0.01	0.05	0.04	0.01	0.07	0.01	0.07	0.01	0.01	nd
M30	0.19	0.12	0.15	0.26	0.07	0.13	0.12	0.03	0.01	0.01	0.02	0.01	0.01	0.04
H31S	0.24	0.14	0.22	0.39	0.10	0.43	0.28	0.04	0.03	0.01	0.04	0.02	0.01	0.03
H31R	0.14	0.08	0.12	0.23	0.06	0.22	0.15	0.02	0.01	0.02	0.02	0.08	0.03	0.01
GAM	0.14	0.16	0.40	1.12	0.05	0.20	0.16	0.06	0.01	0.04	0.02	0.01	0.03	0.05
H32S	1.21	0.01	0.01	0.03	0.02	0.02	0.02	n.d.	n.d.	n.d.	0.02	n.d.	n.d.	n.d.
H32R	n.d.	0.02	0.02	0.03	n.d.	0.04	0.03	0.01	n.d.	n.d.	0.01	n.d.	0.01	0.07
H33S	0.11	0.06	0.09	0.18	0.05	0.18	0.11	0.01	0.01	n.d.	0.02	0.01	0.02	0.08
H33R	0.13	0.04	0.07	0.12	0.04	0.11	0.08	0.01	0.01	n.d.	0.02	0.03	0.02	0.01
H34S	0.13	0.01	0.03	0.06	0.03	0.06	0.05	0.02	n.d.	n.d.	0.01	0.01	0.01	n.d.
H34R	0.02	nd	0.02	0.04	0.01	0.04	0.02	n.d.	n.d.	n.d.	0.01	n.d.	0.01	n.d.
H35S	nd	0.02	0.02	0.03	0.02	0.03	0.02	n.d.	n.d.	n.d.	0.03	n.d.	n.d.	n.d.
H35R	0.02	0.01	0.01	0.03	0.03	0.02	n.d.	n.d.	n.d.	n.d.	0.01	n.d.	n.d.	n.d.
S21	0.06	0.06	0.12	0.17	0.13	0.14	0.14	0.03	0.06	0.01	0.14	0.04	0.02	0.01
S22	0.04	0.04	0.06	0.09	0.02	0.07	0.08	0.02	0.03	0.06	0.08	0.02	0.01	0.01
DIA27S	0.06	0.02	0.02	0.02	0.01	0.02	0.04	0.04	0.05	0.02	0.04	0.04	0.03	0.02
DIA27R	0.04	0.02	0.02	0.03	0.07	0.03	0.04	0.01	0.01	0.03	0.03	0.01	0.01	0.01
DIA27SS	0.07	0.06	0.01	0.09	0.01	0.03	0.04	0.01	0.05	0.02	0.03	0.07	0.02	0.01
DIA27RR	0.02	0.01	0.02	0.03	0.01	0.03	0.02	0.03	0.01	0.02	0.03	0.04	0.01	0.01
C27S	0.08	0.07	0.32	0.73	0.02	0.11	0.10	0.04	0.03	0.01	0.04	0.01	0.03	0.02
C27BBR	0.03	0.04	0.22	0.36	0.04	0.13	0.15	0.02	0.05	0.01	0.09	0.02	0.06	0.03
C27BBS	0.72	0.02	0.14	0.24	0.02	0.09	0.07	0.01	0.02	0.01	0.04	0.01	0.04	0.02
C27R	0.02	0.12	0.31	0.76	0.01	0.07	0.08	0.06	0.02	nd	0.03	0.01	0.03	0.02
C28S	0.01	0.04	0.02	0.03	0.03	0.03	0.03	0.01	0.03	0.01	0.01	0.02	0.03	0.01
C28BBR	0.01	0.01	0.01	0.14	0.02	0.02	0.03	0.01	0.08	0.02	0.02	0.01	0.03	0.02

C28BBS	0.01	0.01	0.05	0.07	0.03	0.04	0.03	0.01	0.01	0.01	0.01	0.01	0.02	0.01
C28R	0.07	0.05	0.18	0.40	0.03	0.03	0.02	0.02	0.01	nd	0.02	0.01	0.02	0.01
C29S	0.01	0.04	0.21	0.36	0.03	0.11	0.04	0.02	0.01	0.03	0.01	0.01	0.03	0.02
C29BBR	0.04	0.03	0.12	0.18	0.01	0.05	0.04	0.01	0.02	0.01	0.02	0.01	0.02	n.d.
C29BBS	0.01	0.01	0.03	0.05	0.01	0.04	0.04	0.01	0.01	0.01	0.01	0.01	0.02	0.01
C29R	0.01	0.01	0.03	0.05	0.01	0.03	0.02	0.01	0.01	n.d.	0.01	n.d.	0.01	0.01
DIA27S	0.063	0.02	0.02	0.02	0.01	0.02	0.14	0.01	0.01	0.01	0.04	0.04	0.03	0.02
DIA27R	0.04	0.02	0.02	0.03	0.01	0.03	0.07	0.01	0.01	0.01	0.03	0.04	0.04	0.04
TPP1	0.01	0.01	0.02	0.03	0.01	0.01	0.02	n.d.	0.01	n.d.	n.d.	n.d.	n.d.	n.d.
TPP2	0.01	0.01	0.02	0.03	0.04	0.02	0.01	0.02	0.01	nd	0.01	nd	0.01	0.01

Table S5. Identified N-markers compounds and their respective concentrations in crude oil samples

N-marker	REC 1	REC 2	POT 1	POT 2	3 MLL	6CHT	9LL	SERG	PAR 1	PAR 2	COL 1	COL 2	VEN 1	VEN 2
	Concentration (mg g^{-1})													
QN	0.02	0.02	0.07	0.08	0.18	0.39	0.25	0.06	0.04	0.05	0.08	0.09	0.09	0.10
IN	0.0022	0.02	0.01	0.01	0.08	0.08	0.04	0.03	0.01	0.02	0.03	0.03	0.04	0.03
4-MQ	0.01	0.01	0.01	0.01	0.04	0.01	0.02	0.01	0.01	0.01	0.01	0.01	0.03	0.01
3-MI	0.01	0.01	0.03	0.03	0.08	0.08	0.05	0.06	0.02	0.09	0.08	0.12	0.02	0.07
2,4-DMQ	0.03	0.02	0.17	0.20	0.22	0.22	0.18	0.24	0.11	1.66	0.39	0.41	0.27	0.22
AC	1.07	4.83	11.2	11.3	15.1	13.3	18.4	16.9	14.8	36.9	25.3	28.3	13.7	11.2
B[c]Q	0.94	5.03	10.2	10.3	13.5	12.4	15.7	18.0	16.1	38.0	24.3	31.9	13.3	10.6
CA	0.01	nd	0.03	0.46	3.74	1.92	3.18	0.08	0.63	1.04	0.79	1.41	0.75	1.82
1-MC	0.01	0.04	0.14	1.37	9.39	6.29	8.68	0.38	3.55	3.13	2.91	2.82	2.75	5.18
3-MC	0.01	0.02	0.03	0.21	4.14	2.61	4.20	0.13	0.88	0.72	1.21	1.47	1.11	1.75
2-MC	0.01	0.01	0.04	0.60	5.11	3.61	4.30	0.16	1.22	1.09	1.20	1.39	1.09	2.01
1,8-DMC	0.01	0.01	0.01	0.05	0.17	1.00	3.64	0.56	0.63	0.44	0.54	1.73	0.55	2.39
1,4-DMC	0.01	0.01	0.01	0.05	0.23	1.24	3.61	3.25	0.66	0.48	0.64	1.64	0.61	2.71
3-EC	0.01	0.01	0.04	0.26	0.74	4.44	12.6	1.50	1.66	1.15	1.64	6.79	2.16	93.0
3,6-DMC	0.01	0.01	0.01	0.03	0.10	0.61	2.46	2.01	0.13	0.08	0.24	2.58	0.17	0.70
2,7-DMC	0.01	0.01	0.01	0.04	0.08	0.56	1.79	0.70	0.16	0.09	0.22	3.33	0.23	0.85
1,4,8-TMC	0.06	0.36	1.56	4.84	30.5	21.6	22.9	2.97	10.9	4.52	9.80	9.57	11.9	16.2
9H CA	0.50	0.50	0.50	0.50	0.50	0.50	0.50	0.50	0.50	0.50	0.50	0.50	0.50	0.50
B[a]C	0.01	0.06	0.21	0.32	0.12	0.81	0.97	0.40	0.16	0.12	0.12	0.14	0.09	0.10
B[b]C	0.01	0.03	0.06	0.07	0.16	0.07	0.12	0.04	0.03	0.04	0.07	0.02	0.06	0.08
B[c]C	0.01	0.01	0.04	0.22	0.73	0.49	0.77	0.04	0.17	0.20	0.11	0.14	0.12	0.24
DBC	nd	0.01	0.01	0.01	0.01	0.01	0.01	0.01	nd	0.01	0.01	0.01	nd	0.01
4-MC	0.01	0.01	0.07	2.01	17.8	3.85	3.52	0.44	1.58	1.47	1.04	1.44	1.15	2.88

1,3-DMC	0.01	0.01	0.01	0.01	0.01	0.04	0.39	4.10	0.03	0.05	0.03	0.45	0.03	0.10
2,4-DMC	0.01	0.01	0.01	0.05	0.03	0.11	0.38	0.09	0.01	0.02	0.03	1.51	0.03	0.17

Table S6. Ratios and concentration of saturated biomarkers for crude oil samples

**Ratio**	**REC 1**	**REC 2**	**POT 1**	**POT 2**	**3 MLL**	**6 CHT**	**9 LL**	**SERG**	**PAR 1**	**PAR 2**	**COL 1**	**COL2**	**VEN 1**	**VEN 2**
TS/TS+Tm	0.53	0.52	0.44	0.38	0.33	0.26	0.26	0.50	0.47	0.67	0.56	0.61	0.34	0.49
dia/dia+C_{27}	0.53	0.56	0.46	0.50	0.50	0.50	0.50	0.50	0.68	0.50	0.50	0.50	0.61	0.58
Total (Tric/Hop)	1.21	1.67	0.96	1.75	4.20	3.24	1.72	1.63	11.58	6.17	7.70	2.01	3.77	7.31
Steranes C_{27}	0.86	0.24	0.99	2.08	0.10	0.40	0.39	0.13	0.19	0.05	0.16	0.09	0.86	0.24
Steranes C_{28}	0.09	0.06	0.35	0.64	0.01	0.12	0.10	0.02	0.06	0.02	0.07	0.04	0.09	0.06
Steranes C_{29}	0.12	0.09	0.38	0.64	0.05	0.23	0.14	0.04	0.04	0.01	0.07	0.03	0.12	0.09

Table S7. Ratios of N-markers in crude oil samples

**Ratio**	**REC 1**	**REC 2**	**POT 1**	**POT 2**	**3 MLL**	**6 CHT**	**9 LL**	**SERG**	**PAR 1**	**PAR 2**	**COL 1**	**COL 2**	**VEN 1**	**VEN 2**
1MC/1MC+3MC	0.65	0.63	0.84	0.87	0.69	0.67	0.71	0.74	0.80	0.81	0.71	0.66	0.71	0.75
4MC/4MC+CA	0.60	0.96	0.73	0.81	0.83	0.52	0.67	0.84	0.72	0.59	0.57	0.51	0.61	0.61
CA	0.00	0.00	0.03	0.46	3.74	3.18	1.92	0.08	0.63	1.04	0.79	1.41	0.75	1.82
ΣBCA	0.02	0.10	0.31	0.61	1.00	1.86	1.37	0.49	0.37	0.35	0.30	0.30	0.26	0.42
4-MC	0.00	0.01	0.07	2.01	17.82	3.52	3.85	0.44	1.58	1.47	1.04	1.44	1.15	2.88
QN	0.01	0.01	0.04	0.09	0.22	0.33	0.32	0.28	0.02	2.78	2.28	0.10	0.39	0.10
4-MQ	0.00	0.00	0.01	0.01	0.50	0.02	0.01	0.09	0.01	0.15	0.02	0.00	0.03	0.03

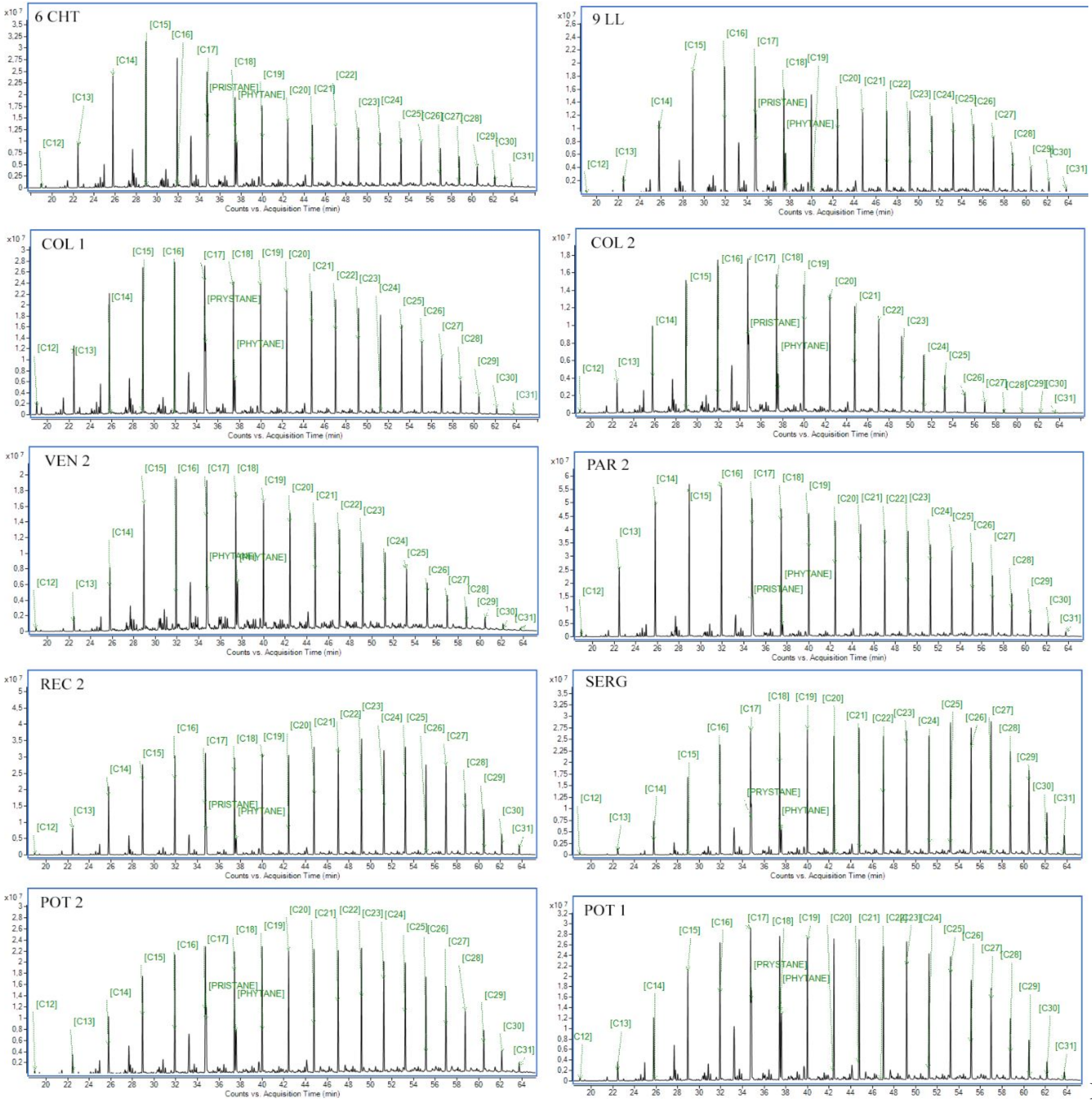

Fig. S1 TPH chromatogram of total oil GC/FID

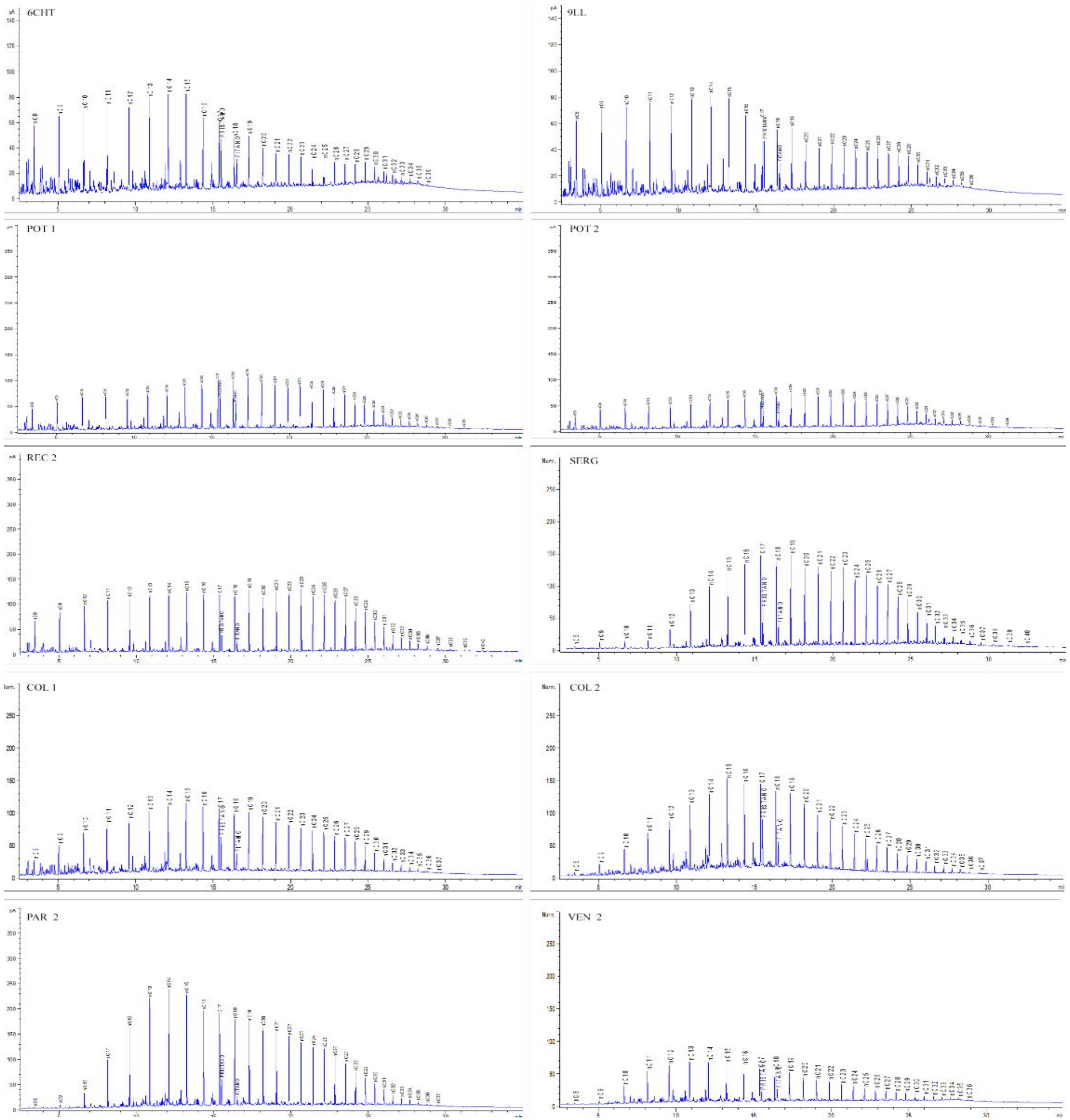

Fig. S2 GC-MS chromatogram of the saturated fractions of oils.

REFERENCES

RANGEL, A.; OSORNO, J.F., RAMIREZ, J.C., DE BEDOUT, J., GONZÁLEZ, J. L.; PABÓN, J.M. Geochemical assessment of the Colombian oils based on bulk petroleum properties and biomarker parameters. Marine and Petroleum Geology, v. 86, p. 1291-1309, 2017.

HWANG, R.J. Biomarker Analysis Using GC-MSD. Journal of Chromatographic Science, v. 28, n. 3, p. 109-113, 1990.

YONEYAM, Y.; YAMAZAKI, M.; KITA, F.Y.O.; YOSHIMOTO, S.; KATO, T. Analysis of the solvent-soluble products of Yubari and Taiheiyo coals treated with zinc and butyl iodide under mild conditions. Analyses of saturate fractions. Journal of the Japan Institute of Energy, v. 75, n. 1, p. 35-41, 1996.
